# RFX6 facilitates aerobic glycolysis‐mediated growth and metastasis of hepatocellular carcinoma through targeting PGAM1

**DOI:** 10.1002/ctm2.1511

**Published:** 2023-12-13

**Authors:** Zhiyu Qiu, Chenwei Wang, Pinzhu Huang, Yichuan Yuan, Yunxing Shi, Zhu Lin, Zhenkun Huang, Dinglan Zuo, Jiliang Qiu, Wei He, Jingxian Shen, Yi Niu, Yunfei Yuan, Binkui Li

**Affiliations:** ^1^ State Key Laboratory of Oncology in South China and Collaborative Innovation Center for Cancer Medicine Sun Yat‐Sen University Cancer Center Sun Yat‐Sen University Guangzhou P. R. China; ^2^ Department of Liver Surgery Sun Yat‐Sen University Cancer Center Sun Yat‐Sen University Guangzhou P. R. China; ^3^ Guangdong Provincial Key Laboratory of Colorectal and Pelvic Floor Disease and Department of Colon and Rectum Surgery The Sixth Affiliated Hospital of Sun Yat‐Sen University Guangzhou P. R. China; ^4^ Department of Radiology Sun Yat‐Sen University Cancer Center Sun Yat‐Sen University Guangzhou P. R. China

**Keywords:** glycolysis, hepatocellular carcinoma, PGAM1, prognosis, RFX6

## Abstract

**Background:**

Hepatocellular carcinoma (HCC) cells undergo reprogramming of glucose metabolism to support uncontrolled proliferation, of which the intrinsic mechanism still merits further investigation. Although regulatory factor X6 (RFX6) is aberrantly expressed in different cancers, its precise role in cancer development remains ambiguous.

**Methods:**

Microarrays of HCC tissues were employed to investigate the expression of RFX6 in tumour and adjacent non‐neoplastic tissues. Functional assays were employed to explore the role of RFX6 in HCC development. Chromatin immunoprecipitation, untargeted metabolome profiling and sequencing were performed to identify potential downstream genes and pathways regulated by RFX6. Metabolic assays were employed to investigate the effect of RFX6 on glycolysis in HCC cells. Bioinformatics databases were used to validate the above findings.

**Results:**

HCC tissues exhibited elevated expression of RFX6. High RFX6 expression represented as an independent hazard factor correlated to poor prognosis in patients with HCC. RFX6 deficiency inhibited HCC development in vitro and in vivo, while its overexpression exerted opposite functions. Mechanistically, RFX6 bound to the promoter area of phosphoglycerate mutase 1 (PGAM1) and upregulated its expression. The increased PGAM1 protein levels enhanced glycolysis and further promoted the development of HCC.

**Conclusions:**

RFX6 acted as a novel driver for HCC development by promoting aerobic glycolysis, disclosing the potential of the RFX6–PGAM1 axis for therapeutic targeting.

## Graphical abstract

Elevated regulatory factor X6 (RFX6) expression serves as an independent prognostic factor associated with poor outcomes in hepatocellular carcinoma (HCC).

RFX6 facilitates the proliferation and motility of HCC cells.

By transcriptionally upregulating phosphoglycerate mutase 1 (PGAM1), RFX6 augments glycolysis and thereby accelerates the progression of HCC.

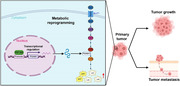

## 1 INTRODUCTION

Hepatocellular carcinoma (HCC) remains a significant contributor to cancer‐associated mortality, ranking third globally.^1^, ^2^ The majority of HCC cases are diagnosed with multiple tumours (*n* > 3), vascular invasion or distant metastasis, leaving a few curative options.^3^ Despite the advances in cancer treatment, current therapeutic options for HCC are limited, emphasising the need to comprehend the molecular mechanisms governing HCC development and identify specific novel therapeutic targets.

In response to demands for sufficient energy and biomolecules, HCC and other solid cancer cells adapt their metabolism in diverse ways, with one well‐known alteration being a shift in glucose metabolism, termed the Warburg effect or aerobic glycolysis.^4^ Apart from its role in energy production and biosynthesis, aerobic glycolysis remodels the tumour microenvironment of HCC, promoting angiogenesis, local invasion and even immune evasion of cancer cells.^5^ Studies targeting key enzymes and regulatory factors have demonstrated the efficacy of suppressing aerobic glycolysis in HCC.^6^, ^7^, ^8^ Therefore, a better understanding of underlying mechanisms will enable researchers to develop new therapies for HCC.

Regulatory factor X6 (RFX6), a winged helix transcription factor, belongs to the regulatory factor X (RFX) family, which is a highly conserved DNA‐binding protein family.^9^ RFX6 expresses exclusively in pancreatic islets and is indispensable to islet cell differentiation.^10^, ^11^ Intriguingly, a genome‐wide association study identified *GPRC6A/RFX6* as a susceptible locus for prostate cancer in a Japanese population, first revealing a link between RFX6 and tumourigenesis.^12^ In subsequent studies, suppression of RFX6 profoundly diminished the development of prostate cancer and HCC, highlighting its pro‐tumoural functions.^13^, ^14^ However, some other researchers obtained opposite results in different malignancies, necessitating further detailed studies to elucidate its precise role in HCC.^15^, ^16^

Phosphoglycerate mutase 1 (PGAM1), an instrumental glycolytic enzyme, was identified as one of the downstream targets of RFX6 in the current study. During glycolysis, this enzyme catalyses the switch of 3‐phosphoglycerate (3‐PG) to 2‐phosphoglycerate (2‐PG) and produces a 2,3‐bisphosphoglycerate intermediate, which in turn reactivates itself and facilitates releasing 2‐PG.^17^ Simultaneously, PGAM1 also regulates glycolytic intermediates utilised as precursors for anabolic biosynthesis.^18^ Recent studies have demonstrated aberrant expression of PGAM1 in various cancers, including HCC, and its association with poor clinical outcomes, implying the potential role of PGAM1 in cancer development.^19^, ^20^, ^21^, ^22^, ^23^, ^24^ It was also implicated in DNA double‐strand break repair.^25^ Apart from glucose metabolism, this glycolytic enzyme also exacerbates cancer cell migration via modulating the assembly of actin filaments.^26^ Therefore, PGAM1 might predominantly mediate the pro‐tumoural functions of RFX6 in HCC.

In current study, we revealed the high expression of RFX6 in HCC patients, which indicated poor clinical outcomes. We illustrated the pro‐tumoural role of RFX6 in HCC through loss and gain‐of function assays. RFX6 facilitates glycolysis via transcriptionally upregulating PGAM1, further promoting the growth and metastasis of HCC. Our results uncovered a novel RFX6–PGAM1 axis in HCC progression with potential therapeutic implications.

## 2 METHODS

### 2.1 Patients and specimens

A total of 125 patients who underwent curative surgery at the Sun Yat‐Sen Cancer Center (SYSUCC; Guangzhou, China), between January 2010 and May 2015, were included in the present study. Matched tumour and normal tissue samples of included patients were collected for constructing a tissue microarray. Table S1 presents the clinicopathological characteristics of included patients. Overall survival (OS) was defined as the time interval between the date of death or last follow‐up, and the date of radical surgery. Disease‐free survival (DFS) was defined as the time interval between the date of tumour recurrence or last follow‐up, and the date of radical surgery. The informed consent was obtained from all included patients.

### 2.2 Cell lines and cell culture

Cells were maintained as described previously.^27^ Please refer to the part of materials and methods in the Supporting Information.

### 2.3 Immunohistochemistry and immunofluorescent staining

Immunohistochemistry (IHC) staining was performed as described previously.^28^, ^29^ Particularly, HALO (Indica Labs) was adopted to review and score IHC sections in this study. Based on the area quantification method, a staining index was obtained as the intensity of staining (negative = 0, weak = 1, moderate = 2, strong = 3 scores) and the proportion of immunopositive cells of interest (≤10% = 1, >10% to ≤50% = 2, >50% to ≤75% = 3, >75% = 4 scores) were calculated. Based on the scores, patients in this experimental cohort were dichotomised as negative group, 6 ≤ score < 7; weak group, 7 ≤ score < 9; moderate group, 9 ≤ score < 11; strong group, 11 ≤ score. RFX6‐high expression group was defined as patients with tumours of strong or moderate intensities, while RFX6‐low expression group was defined as patients with tumours of weak or negative intensities. For more details, please refer to the part of materials and methods in the Supporting Information.

Immunofluorescent (IF) staining was performed by using tyramide signal amplification‐conjugated fluorophores to detect targets. Tissue specimen slides were probed with the following primary antibody: anti‐RFX6 antibody (Proteintech, 22551‐1‐AP, 1:200 dilution). For more details, please refer to the part of materials and methods in the Supporting Information.

### 2.4 Luciferase reporter and chromatin immunoprecipitation assay

Luciferase reporter and chromatin immunoprecipitation (ChIP) assay were performed as described previously.^30^ For more details, please refer to the part of materials and methods in the Supporting Information.

### 2.5 Seahorse experiments

Seahorse XF assays were conducted to measure the extracellular acidification rate (ECAR) as a measure of glycolysis. Briefly, cells were seeded and analysed in 24‐well XF Cell Culture Microplates (8 × 10^4^ cells per well) as described previously.^31^

### 2.6 Fluoro‐2‐D‐deoxyglucose F18‐PET imaging and analysis

Mice were fasted for 12 h before positron emission tomography–computed tomography (PET–CT) scanning and received fluoro‐2‐D‐deoxyglucose F18 ([^18^F]‐FDG) (radiochemical purity ≥95%, Shanghai Atomic Kexing Pharmaceutical Co., Ltd., max. 100 μCi) injection via the coccygeal vein. After resting 1 h, mice underwent PET–CT scans using a micro‐PET/CT scanner (Siemens) under anesthesia. Standardised uptake value (SUV) was obtained from the PET/CT workstation as a quantification of FDG uptake in the regions of interest on tumour and adjacent areas. A 30% of the maximum uptake value in volumes of interest was considered as a low threshold to differentiate the tumour boundary. A nuclear medicine physician was responsible for the identification and measurement of SUV maximum (SUV_max_) values. Relative SUV_max_ was defined as dividing the SUV_max_ of the tumour by that of the adjacent area.

### 2.7 Animal models

Subcutaneous tumour models, xenograft tumour models and lung metastasis models were established as described previously.^32^

### 2.8 Lactate and 2‐PG production assay

Lactate and 2‐PG production were measured according to manufacturer's instructions. HCC cells were first seeded in 6 cm dishes at a density of 2 × 10^6^ cells per dish and cultured with complete medium for 48 h in a 37°C incubator.

For lactate production assay (Nanjing Jiancheng Bioengineering Institute), cells were prepared with ice cold phosphate buffered saline on ice. Then samples were centrifuged at 12 000 rpm for 5 min to obtain the supernatant. Reaction mix was prepared and added to each sample according to the instruction. Then, the mixture was incubated at 37°C for 10 min and the reaction was terminated. Mixture (250 μL) was added into a 96‐well plate and then measured at 530 nm. The lactate production was calculated based on previous established standard curve.

For 2‐PG production assay (Abnova), 1 × 10^6^ cells were prepared with 200 μL ice cold 2‐PG assay buffer. Then, samples were centrifuged at 12 000 rpm for 5 min to obtain the supernatant. Supernatants (1−50 μL) were added into a 96‐well plate and the volume could be brought to 50 μL with 2‐PG assay buffer. An amount of 50 μL of reaction mix or background control mix was added to each well and the mixture was then incubated at room temperature for 40 min. The mixture was measured at 570 nm and the 2‐PG production was calculated based on previous established standard curve.

### 2.9 Metabolites extraction from tissue samples

Physiological saline solution was added to the tumour tissues at a ratio of 9 mL:1 g (≤300 mL per tube). After grinding with magnetic beads at 4°C for 2 min, samples were further centrifuged for 10 min (2500 rpm, 4°C) to obtain supernatant (about 10% of added saline). Based on the results of pilot studies, the supernatant was diluted with double distilled water in a ratio of 1: 8 for determining metabolite levels.

### 2.10 Statistical analysis

The experiments were required to repeat independently three times at least. Measurements were mainly represented as mean ± standard deviation. Student's *t*‐test and the Wilcoxon matched pair test were adopted to analyse the quantitative variables. The chi‐squared test was adopted to analyse the qualitative variables. For each statistical analysis, the sample size (*n*) was denoted in figure legends. The upper quartiles of gene expression values were chosen as cut‐off values to separate the RFX6‐high and low‐groups for The Cancer Genome Atlas (TCGA), International Cancer Genome Consortium (ICGC) and National Center for Biotechnology Information Gene Expression Omnibus (GEO) datasets. For survival analysis, OS, DFS and progression‐free survival were calculated and compared utilising the Kaplan–Meier method and the log‐rank test. The prognostic value of each potential risk factor was assessed and screened utilising the Cox proportional hazard regression model. Variables with *p*‐values <.10 in univariate analysis were then input into multivariate analysis. Spearman test was used to assess correlations between qualitative variables. All analyses conducted in this study were two‐tailed, and differences with *p*‐values <.05 were considered significant. R program and GraphPad Prism 9.0 software (GraphPad Software) were used for all statistical analyses .

## 3 RESULTS

### 3.1 Upregulation of RFX6 in HCC tissues and its correlation with poor prognosis

**FIGURE 1:**
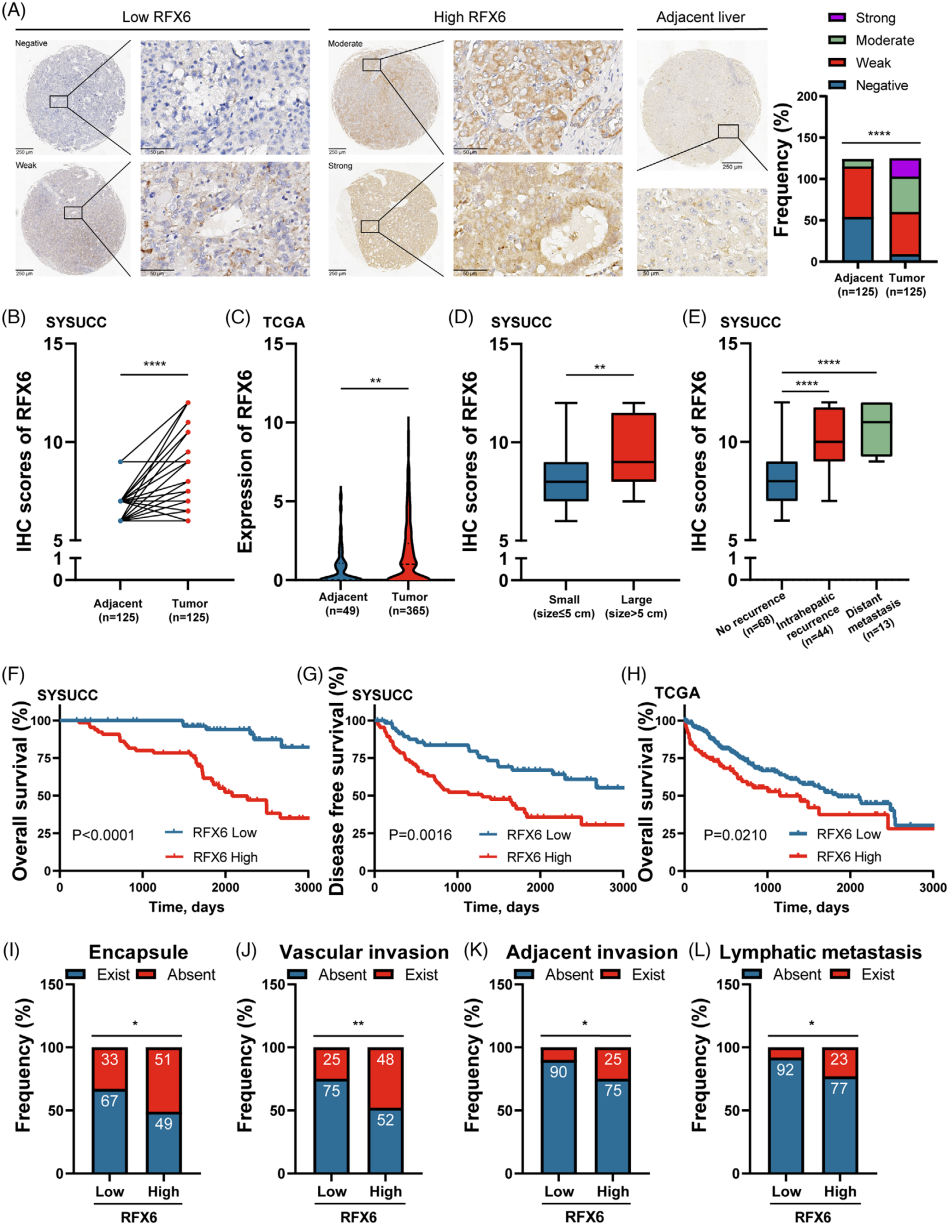
Upregulation of regulatory factor X6 (RFX6) in hepatocellular carcinoma (HCC) tissues and its correlation with poor prognosis. (A) Representative images of different levels of RFX6 expression in HCC (4×, left panel; 40×, right panel) and adjacent noncancerous liver tissues from the Sun Yat‐Sen Cancer Center (SYSUCC) cohort (*n* = 125) (4×, top panel; 40×, bottom panel) detected by immunohistochemical staining (IHC) are shown. Stacked bar plots represent the RFX6 expression patterns in tumour and adjacent tissues. Scale bar, 250 μm (4×), 50 μm (40×). (B) Comparison of RFX6 expression in HCC and paired noncancerous liver tissues based on the IHC scores in the SYSUCC cohort using the Wilcoxon matched pairs test. (C) Comparison of RFX6 expression in HCC (*n* = 365) and normal tissues (*n* = 49) using the Cancer Genome Atlas (TCGA) data. (D) Comparison of RFX6 expression in large and small HCC tissues based on the IHC scores in the SYSUCC cohort. (E) Comparison of RFX6 expression in HCC tissues with distant metastasis, intrahepatic recurrence and no recurrence based on the IHC scores in the SYSUCC cohort. (F) Overall survival (OS) curves for patients with high and low RFX6 expression in the SYSUCC cohort. (G) Disease‐free survival (DFS) curves for patients with high and low RFX6 expression in the SYSUCC cohort. (H) OS curves for patients with high and low RFX6 expression using the TCGA data (*n* = 365). (I) Distribution of absent and existing encapsule in the RFX6‐low and RFX6‐high groups. (J) Distribution of existing and absent vascular invasion in the RFX6‐low and RFX6‐high groups. (K) Distribution of existing and absent adjacent invasion in the RFX6‐low and RFX6‐high groups. (L) Distribution of existing and absent lymphatic metastasis in the RFX6‐low and RFX6‐high groups. Upper quartiles of IHC score and gene expression were chosen as cut‐off values for distinguishing between the RFX6‐low and RFX6‐high groups. ^*^ *p* < .05, ^**^ *p* < .01 and ^****^ *p* < .0001.

A tissue microarray from the SYSUCC cohort was employed to investigate whether RFX6 was clinically meaningful in the development of HCC. Determined by IHC staining, the protein expression of RFX6 was significantly elevated in HCC tissues over adjacent noncancerous tissues (*p* < .0001) (Figure 1A,B), which was substantiated by the mRNA levels from TCGA, ICGC and GEO (Figures 1C and S1A–D). IF staining was further conducted to confirm the subcellular localisation of RFX6 in HCC tissues (Figure S1E). Clinicopathologically, patients with large tumours exhibited higher IHC scores compared to those with small tumours (Figure 1D). Moreover, RFX6 expression displayed a stepwise increase from no recurrence to distant metastasis stage, linking to tumour aggressiveness (Figure 1E).

Based on the IHC scores, patients were categorised into RFX6‐high and RFX6‐low expression groups. On survival analysis, the RFX6‐high expression group exhibited worse OS and DFS than the RFX6‐low expression group, which was also substantiated by public datasets (Figures 1F–H and S1F,G). In the GSE54236 dataset, the RFX6‐high expression group were more likely to double the tumour size than the RFX6‐low expression group (Figure S1H). Furthermore, the chi‐squared test revealed that upregulated RFX6 expression was correlated to the absence of tumour capsules, vascular invasion, adjacent organ invasion and lymphatic metastasis (Figure 1I–L and Table S1). Upon Cox regression analyses, upregulated RFX6 represented an independent prognostic factor for OS and DFS (Tables S2 and S3). Accumulatively, the above results suggest that high RFX6 expression predicts poor prognosis and aggressive clinicopathological characteristics in HCC patients.

### 3.2 Silencing RFX6 suppresses the proliferation and motility of HCC cells in vitro and in vivo

**FIGURE 2:**
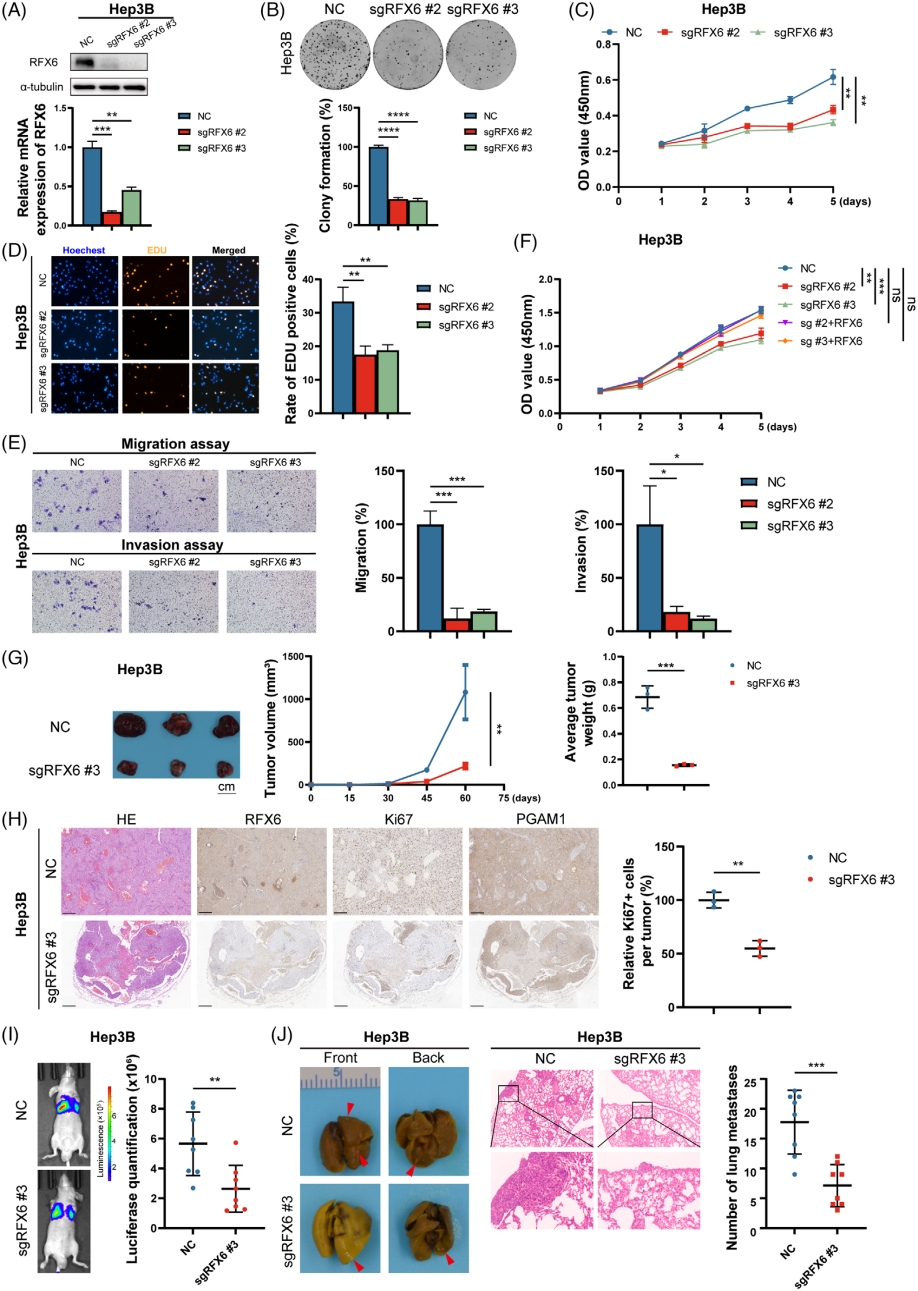
Silencing regulatory factor X6 (RFX6) suppresses the proliferation and motility of hepatocellular carcinoma (HCC) cells in vitro and in vivo. (A) Western blotting (top panel) and real‐time polymerase chain reaction (PCR) (bottom panel) demonstrate the efficiency of RFX6‐knockout (KO). (B) RFX6‐KO suppressed the proliferation of HCC cells, as shown by the colony formation assay. (C) RFX6‐KO suppressed the proliferation of HCC cells, as shown by the Cell Counting Kit‐8 (CCK‐8) assay. (D) RFX6‐KO suppressed the proliferation of HCC cells, as shown by the 5‐Ethynyl‐2'‐deoxyuridine (EDU) assay. (E) RFX6‐KO suppressed the migration and invasion capabilities of HCC cells, as shown by the transwell assay. A total of 3.2 × 10^5^ cells were seeded in a chamber, and incubated for 12 h (migration assay) or 36 h (invasion assay). (F) Overexpression of RFX6‐wildtype rescued cell proliferation in Hep3B cells after silencing RFX6, as shown by the CCK‐8 assay. (G) RFX6‐KO suppressed HCC growth, as indicated by tumour volumes and weights (*n* = 3/group). Scale bar, 1 cm. (H) Haematoxylin/eosin and immunohistochemical staining of RFX6, Ki67 and downstream protein in subcutaneous tumours of mice. RFX6‐KO suppressed HCC growth, as indicated by the quantitation of Ki67+ stained cells. Scale bar, 250 μm. (I and J) RFX6‐KO suppressed the lung metastatic capacity of HCC cells, as shown by bioluminescent imaging (I) and quantitation of metastatic nodules (J) (*n* = 8/group). Data are represented as mean ± standard deviation (SD) from at least three independent experiments. A two‐tailed Student's *t*‐test was used for statistical analysis. ^*^ *p* < .05, ^**^ *p* < .01 and ^***^ *p* < .001.

The expression of RFX6 in HCC cell lines was elucidated to investigate the role of RFX6 in HCC progression and metastasis. Due to the high expression of RFX6, Hep3B and PLC/PRF/5 cells were selected to construct stable RFX6‐knockout (KO) and ‐knockdown (KD) cell lines. Two independent small guide RNAs and two small interfering RNAs were separately used for knocking out and knocking down RFX6. The KO and KD efficiency of RFX6 in these cell lines were verified at mRNA and protein levels (Figures 2A and S2A–C). As shown in Figures 2B–D and S2D–F, silencing of RFX6 significantly attenuated HCC cell proliferation. In the transwell assay, silencing of RFX6 diminished HCC cell motility (Figures 2E and S2G,H). Reintroducing RFX6‐overexpressing (OE) plasmids into RFX6‐KO cells reversed the inhibited cell proliferation, corroborating the instrumental role of RFX6 in HCC cells (Figure 2F).

To characterise and validate the pro‐tumoural function of RFX6 in vivo, nude mice were injected with RFX6‐KO Hep3B cells or their relevant negative controls (NCs) to generate subcutaneous HCC tumour models and in vivo lung metastasis models. In the subcutaneous models, the comparison of growth curves and tumour weights found that RFX6‐KO markedly inhibited the xenograft tumour growth (Figure 2G). IHC staining of the tumour tissue revealed a marked reduction in Ki67+ cells by silencing RFX6 (Figure 2H). In the in vivo lung metastasis models, incidences of lung metastases were reduced in the RFX6‐KO group compared to the NC group, as reflected by reduced luciferase signal and metastatic nodes (Figure 2I,J). Succinctly, silencing RFX6 significantly inhibits HCC cell proliferation and motility both in vitro and in vivo.

### 3.3 Overexpressing RFX6 promotes proliferation and motility of HCC cells in vitro and in vivo

**FIGURE 3:**
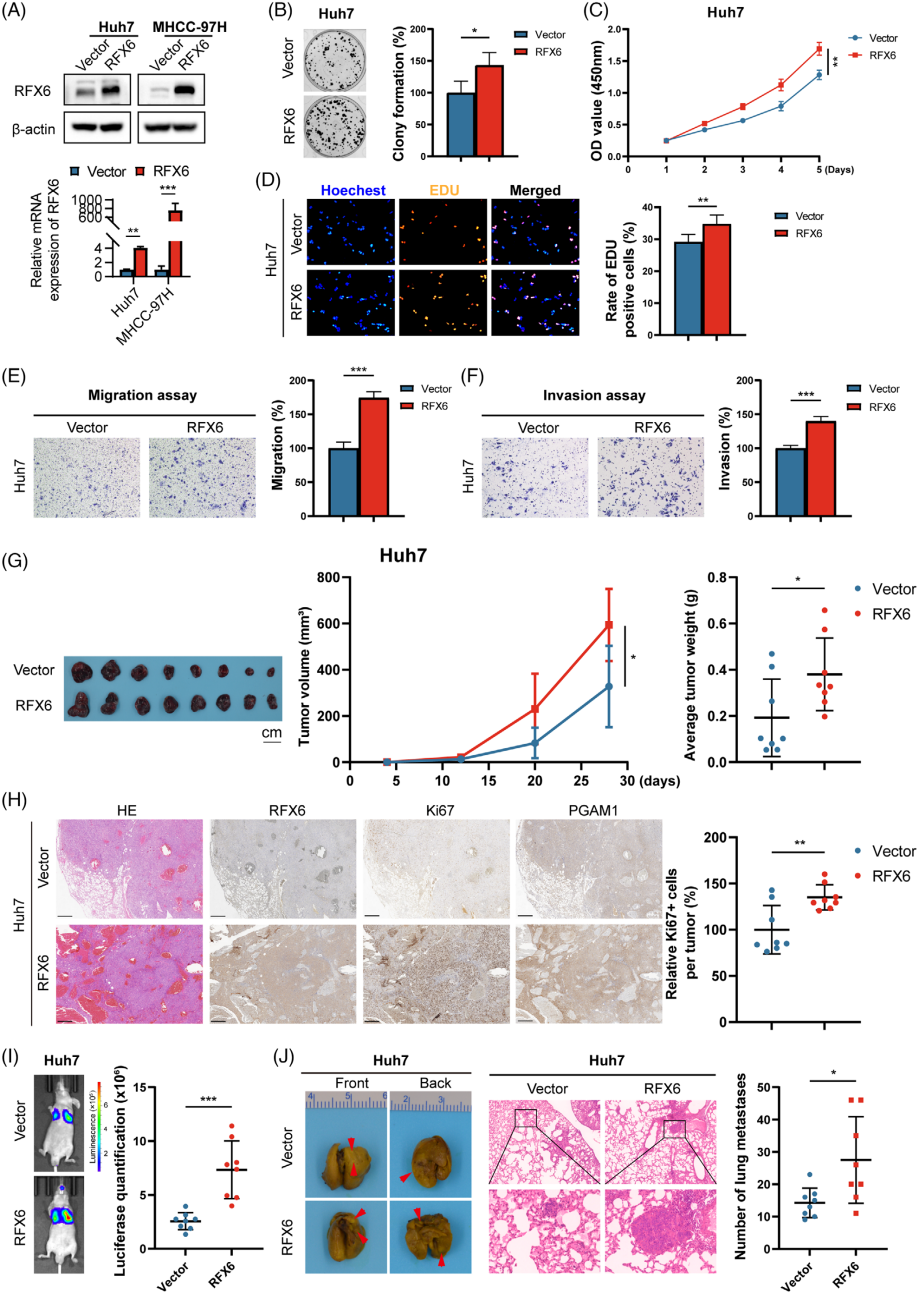
Overexpressing regulatory factor X6 (RFX6) promotes proliferation and motility of hepatocellular carcinoma (HCC) cells in vitro and in vivo. (A) Western blotting (top panel) and real‐time polymerase chain reaction (PCR) (bottom panel) demonstrate the efficiency of RFX6‐overexpressing (OE). (B) RFX6‐OE promoted the proliferation of HCC cells, as shown by the colony formation assay. (C) RFX6‐OE promoted the proliferation of HCC cells, as shown by the Cell Counting Kit‐8 (CCK‐8) assay. (D) RFX6‐OE promoted the proliferation of HCC cells, as shown by the 5‐Ethynyl‐2'‐deoxyuridine (EDU) assay. (E and F) RFX6‐OE promoted the migration (E) and invasion (F) capabilities of HCC cells, as shown by the transwell assay. A total of 1.6 × 10^5^ cells were seeded in a chamber, and incubated for 12 h (migration assay) or 36 h (invasion assay). (G) RFX6‐OE promoted HCC growth, as indicated by tumour volumes and weights (*n* = 8/group). Scale bar, 1 cm. (H) Haematoxylin/eosin and immunohistochemical staining of RFX6, Ki67 and downstream protein in subcutaneous tumours of mice. RFX6‐OE promoted HCC growth, as indicated by the quantitation of Ki67+ stained cells. (I and J) RFX6‐OE enhanced the lung metastatic capacity of HCC cells, as shown by bioluminescent imaging and quantitation of metastatic nodules (*n* = 8/group). Data are represented as mean ± standard deviation (SD) from at least three independent experiments. A two‐tailed Student's *t*‐test was used for statistical analysis. ^*^ *p* < .05, ^**^ *p* < .01 and ^***^ *p* < .001.

To further validate the biological functions of RFX6, Huh7 and MHCC‐97H cells were chosen to construct stable RFX6‐OE cells (Figure 3A). Compared to the vector control groups, the RFX6‐OE group exhibited enhanced cell proliferation (Figures 3B–D and S3A–C). Similarly, the transwell assay showed that RFX6‐OE increased the HCC cell motility (Figures 3E,F and S3D). In the subcutaneous tumour model, the RFX6‐OE group had larger tumour volumes and weights than the vector group (Figure 3G). In a subsequently constructed orthotopic xenograft model, the RFX6‐OE group showed a larger tumour burden than the vector group (Figure S3E,F). IHC staining of the tumour tissues also demonstrated an increased number of positive cells for Ki67 in the RFX6‐OE group (Figures 3H and S3G). In the in vivo lung metastasis model, increased luciferase signals and numbers of metastatic nodes were detected in the RFX6‐OE group (Figure 3I,J). Together, these results suggest that RFX6‐OE enhances the HCC cell proliferation and motility both in vitro and in vivo.

### 3.4 RFX6 promotes aerobic glycolysis in HCC

**FIGURE 4:**
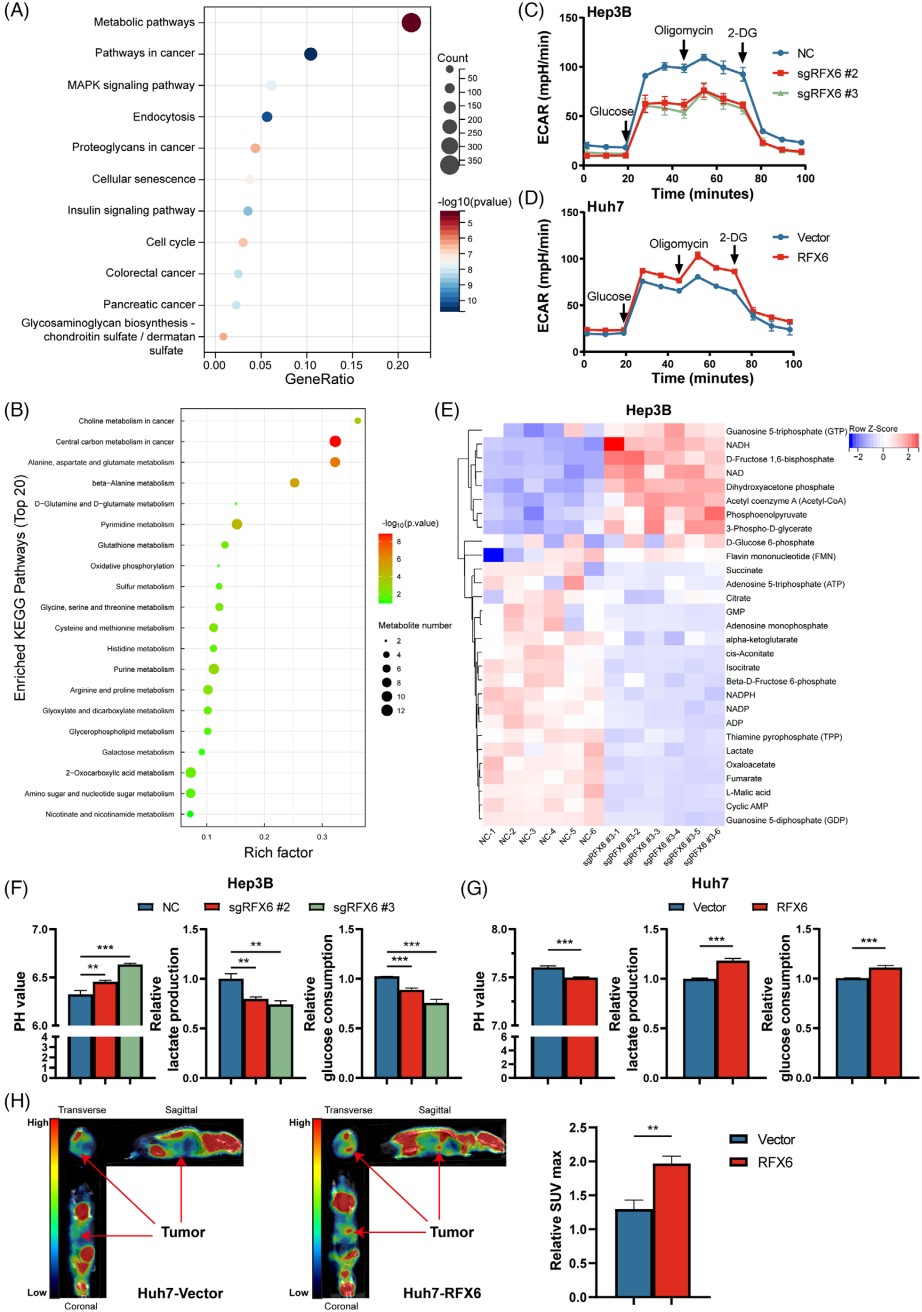
Regulatory factor X6 (RFX6) promotes aerobic glycolysis in hepatocellular carcinoma (HCC). (A) Kyoto Encyclopedia of Genes and Genomes (KEGG) analysis of genes annotated from chromatin immunoprecipitation sequencing (ChIP‐seq) peaks reveals enriched pathways. (B) KEGG analysis of different metabolites identifies enriched pathways. (C) Seahorse experiments determined the extracellular acidification rate (ECAR) in RFX6‐knockout (KO) and control Hep3B cells. (D) Seahorse experiments determined the ECAR in RFX6‐overexpressing (OE) and control Huh7 cells. (E) Heatmap shows glycolysis‐related metabolites in RFX6‐KO and control Hep3B cells. (F) pH of culture medium, lactate production and glucose consumption were measured in RFX6‐KO and control Hep3B cells. (G) pH of culture medium, lactate production and glucose consumption were measured in RFX6‐OE and control Huh7 cells. (H) Representative [^18^F]‐FDG micro‐ positron emission tomography/computed tomography (PET/CT) images of tumour‐bearing mice (left panel) and comparison of relative SUV_max_ between RFX6‐OE and control groups (right panel). Tumours are indicated with arrows. Mice were fasted for 12 h before detection (*n* = 3/group). Data are represented as mean ± standard deviation (SD) from at least three independent experiments. A two‐tailed Student's *t*‐test was used for statistical analysis. ^*^ *p* < .05, ^**^ *p* < .01 and ^***^ *p* < .001.

Since RFX6 is a transcription factor, ChIP followed by next‐generation sequencing analysis (ChIP‐seq) was performed to identify unique peaks from RFX6‐OE MHCC‐97H cells. Kyoto Encyclopedia of Genes and Genomes (KEGG) analysis recognised ‘metabolic pathways’ as one of the significantly enriched pathways (Figure 4A). Untargeted metabolome profiling was performed using mass spectrometry to identify key metabolites to investigate the effect of RFX6 on metabolism (Table S4). Principal components analysis (PCA) exhibited clear separation between RFX6‐KO Hep3B cells and their relevant controls, as well as consistency in the triplicates of each group (Figure S4A). Inspiringly, KEGG pathway analysis showed that metabolites seen in various levels between the two groups were significantly enriched in the central carbon metabolism in cancer pathway (Figure 4B), consisting of glycolysis/glycogenesis and oxidative phosphorylation. Further, transcriptome sequencing was performed in RFX6‐KO cells and relevant controls (Table S5). Gene set enrichment analysis showed that glycolysis and gluconeogenesis pathway was significantly deregulated in RFX6‐KO HCC cells (Figure S4B). We further conducted an ensemble mode analysis to integrate ChIP‐seq and RNA‐seq data by Cistrome‐GO (http://go.cistrome.org/).^33^ This integrative pathway analysis also verified that ‘metabolic pathways’ was one of the top 10 enriched pathways (Table S6). Collectively, these results indicated a potential role of RFX6 in glucose metabolism.

To confirm the influence of RFX6 on glucose metabolism, Seahorse XF experiments were conducted to determine the ECAR levels of HCC cells with different RFX6 expressions. The measures of glycolysis were markedly decreased in RFX6‐silenced cells, whereas both were elevated in RFX6‐OE cells (Figures 4C,D and S4C–F). Metabolome profiling targeting glucose metabolism was further performed to identify key metabolites (Table S7). Significant differences in glycolytic and mitochondrial metabolites between RFX6‐KO Hep3B cells and relevant controls were seen on the metabolomic heatmap (Figure 4E). PCA also exhibited clear separation between the two groups as well as the consistency in the duplicates of each group (Figure S4G). Among metabolites, lactate, a representative product of glycolysis, was significantly reduced in RFX6‐KO cells. Several intermediate products of glycolysis were also accumulated in KO cells, reflecting the deceleration of glycolytic flux (Figure S4H). Collectively, these results revealed the critical role of RFX6 in HCC glycolysis.

Further, pH of the culture medium, lactate production and glucose consumption in RFX6‐KO, ‐KD and ‐OE cells, were measured separately. These assays exhibited that silencing RFX6 reduced the lactate production and the glucose consumption while increased the pH of the culture medium in HCC cells (Figures 4F and S4I). In contrast, overexpressing RFX6 enhanced the lactate production and the glucose consumption while decreased the pH of the culture medium in HCC cells (Figures 4G and S4J). In previously constructed xenograft models, the impact of RFX6 on glucose consumption of HCC cells in vivo was detected by a [^18^F]‐FDG PET–CT scanning. As shown in Figure 4H, tumours of the RFX6‐OE group exhibited an evidently higher level of [^18^F]‐FDG uptake than the control group, illustrating that RFX6 promoted the glucose consumption in HCC cells in vivo. Furthermore, metabolome profiling targeting glucose metabolism was conducted in tumours derived from subcutaneous models and notable fluctuations in glycolytic metabolites were revealed (Table S8). Specifically, glucose and lactate levels were significantly diminished in tumours from the RFX6‐KO group while being elevated in the RFX6‐OE group, which was consistent with previous results (Figure S5A,B). Consequently, manipulation of RFX6 could modify the glycolysis in HCC cells.

To confirm whether the Warburg effect was responsible for the progression of HCC induced by RFX6, RFX6‐OE cells and relevant controls were treated with 2‐deoxyglucose (2‐DG), a glycolysis inhibitor, at different concentrations of 0, 4 and 8 mM for 24 h. The lactate production assay found that 2‐DG significantly inhibited the glycolysis in HCC cells at the concentration of 8 mM (Figure S5C,D). As demonstrated by the Cell Counting Kit‐8 (CCK‐8) and transwell assays, HCC cell proliferation and motility induced by RFX6 were reversed by the treatment of 2‐DG at the concentration of 8 mM (Figure S5E–H). Furthermore, the supplementation of lactate partly rescued the suppression of cell proliferation due to silencing RFX6 (Figure S5I). These results corroborate that RFX6 increases aerobic glycolysis in HCC cells both in vitro and vivo, therefore promoting tumour progression.

### 3.5 RFX6 transcriptionally regulates PGAM1

**FIGURE 5:**
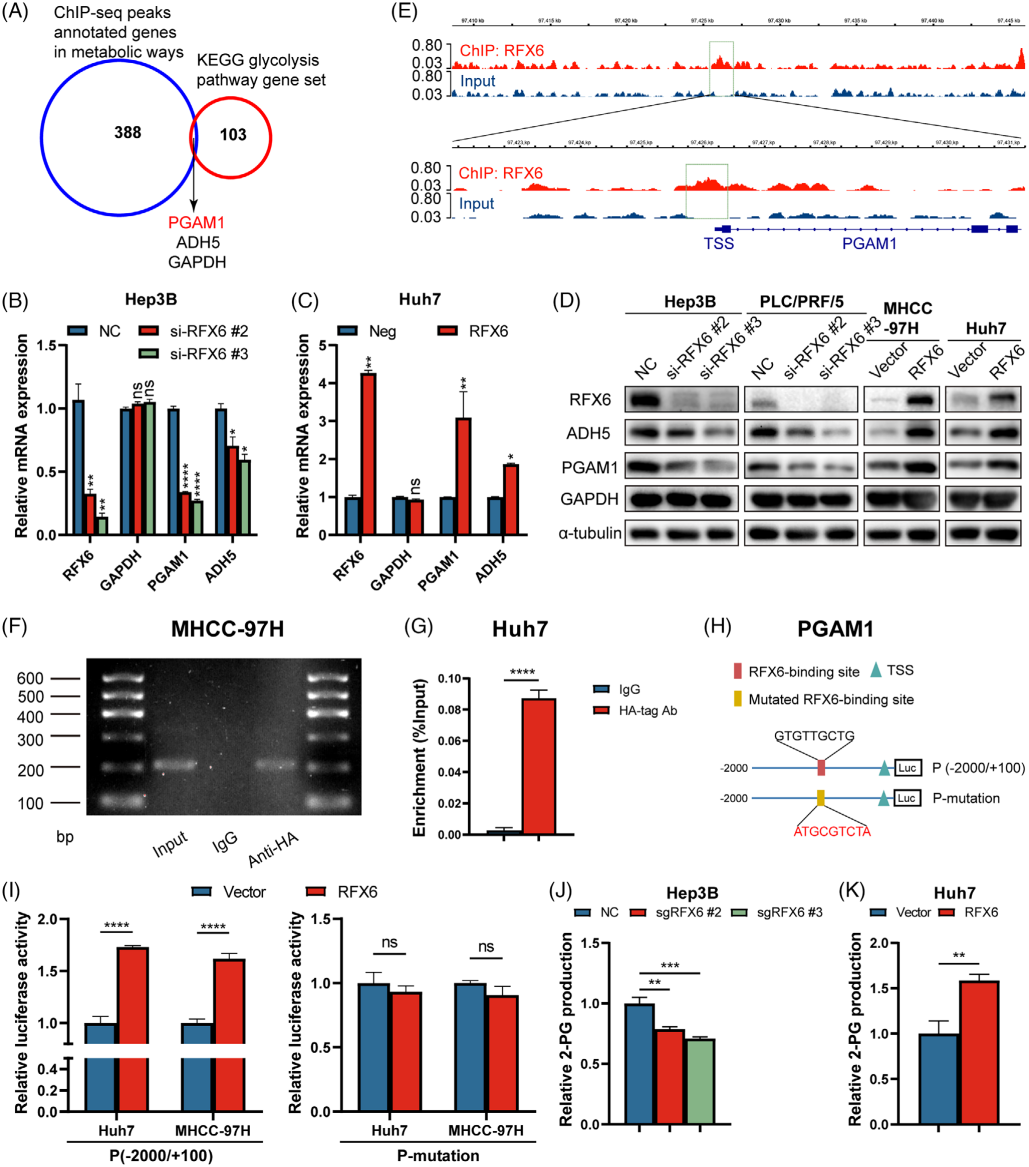
Regulatory factor X6 (RFX6) transcriptionally regulates phosphoglycerate mutase 1 (PGAM1). (A) Venn diagram illustrates the overlap between chromatin immunoprecipitation sequencing (ChIP‐seq) annotated genes from metabolic pathways and the glycolysis pathway gene set in the Kyoto Encyclopedia of Genes and Genomes (KEGG) database. (B) Expression of potential downstream target genes was measured by real‐time polymerase chain reaction (PCR) in RFX6‐knockdown (KD) hepatocellular carcinoma (HCC) cells. (C) Expression of potential downstream target genes was measured by real‐time PCR in RFX6‐overexpressing (OE) HCC cells. (D) Expression of potential downstream target proteins was measured by Western blotting in RFX6‐KD and RFX6‐OE HCC cells. (E) RFX6 occupancy at the PGAM1 promoter visualised by the Integrated Genomics Viewer. (F) PGAM1 is transcriptionally regulated by RFX6 in MHCC‐97H cells, as shown by ChIP‐semiquantitative PCR. Immunoglobulin G (IgG) served as the control. (G) ChIP‐qPCR analysis of RFX6 occupancy at the PGAM1 promoter region in Huh7 cells. IgG served as the control. (H) Schematic illustration of the putative RFX6 binding site in P (−2000/+100) and the corresponding mutated sequence in P‐mutation. (I) PGAM1 promoter luciferase activity in Huh7 or MHCC‐97H cells. RFX6‐OE increased the luciferase activity of P (−2000/+100) (left panel). RFX6‐OE did not affect the luciferase activity of P‐mutation (right panel). HCC cells were reverse‐transfected with either the vector or RFX6‐OE plasmid for 24 h, followed by transfection with P (−2000/+100) or P‐mutation for another 48 h, and then subjected to luciferase activity analysis. (J) 2‐Phosphoglycerate (2‐PG) production was measured in RFX6‐knockout and control Hep3B cells. (K) 2‐PG production was measured in RFX6‐OE and control Huh7 cells. Data are represented as mean ± standard deviation (SD) from at least three independent experiments. ^*^ *p* < .05, ^**^ *p* < .01, ^***^ *p* < .001 and ^****^ *p* < .0001.

Considering RFX6 as a promoter of glycolysis, intersecting genes were considered between ChIP‐seq peaks annotated genes enriched in metabolic pathways and glycolysis pathway gene set from the KEGG database (https://www.kegg.jp/, hsa00010). *PGAM1*, *ADH5* and *GAPDH* were identified as the target genes for RFX6 in regulating glycolysis activation (Figure 5A). Further examination of mRNA and protein expression in the RFX6‐KD and ‐OE cells found that only PGAM1 and ADH5 displayed similar changes both in mRNA and protein levels (Figures 5B–D and S6A,B). Overexpressing or silencing RFX6 could enhance or repress the expressions of PGAM1 and ADH5, indicating that RFX6 might transcriptionally regulate these two genes. Notably, manipulation of RFX6 expression levels influenced the expression of PGAM1 more than ADH5. Visual examination of the above ChIP‐seq data illustrated that, as expected, promoters of these two genes were enriched for RFX6 (Figures 5E and S6C). Further examination of the upstream regions of PGAM1 and ADH5 (−2000/+100 kb) in a public database GPMiner (http://gpminer.mbc.nctu.edu.tw/) identified putative RFX6 binding sites in their promoters. Since the RFX6 primary antibody used in the current study was not suitable for ChIP assays, we constructed HA‐tagged RFX6‐OE plasmids for ChIP assays. To verify the physical interaction between RFX6 and these promoters, ChIP assays were performed by transducing HA‐tagged RFX6‐OE plasmids in MHCC‐97H (ChIP‐semiquantitative polymerase chain reaction [PCR]) and Huh7 (ChIP‐qPCR) cells (Figure S6D). ChIP‐semiquantitative PCR demonstrated that RFX6 could bind to the putative site of the PGAM1 promoter region rather than that of the ADH5 promoter region, indicating the physical interaction of RFX6 with the promoter of PGAM1 (Figures 5F,G and S6E).

To further investigate the functional significance of RFX6‐mediated regulation of PGAM1, luciferase plasmids with either wildtype or binding site‐mutated PGAM1 promoter sequences were constructed (Figure 5H). Increased luciferase activity of the wildtype construct was seen when RFX6 was overexpressed, whereas mutation of the binding site abrogated the RFX6‐mediated potentiation of promoter reporter activity (Figure 5I). These results suggested the recruitment of endogenous or exogenous RFX6 to the putative binding site of the PGAM1 promoter region. Furthermore, the expressions of RFX6 and PGAM1 were positively correlated in this experimental cohort and public data (Figure S6F,G). IHC staining of tumour tissues from previous models also showed the decreased expression of PGAM1 in the RFX6‐KO cells and increased expression in the RFX6‐OE cells in vivo (Figures 2H and 3H), supporting the transcriptional regulation of PGAM1 by RFX6 in HCC cells.

In the Hep3B cell line, silencing PGAM1 significantly attenuated cell proliferation and motility (Figure S6H–K), validating its pro‐tumoural function. Since this enzyme catalyses the conversion of 3‐PG to 2‐PG, the metabolome profiling data were retrospectively analysed and several glycolytic metabolites upstream of PGAM1, including 3‐PG, D‐fructose‐1‐6‐bisphosphate and dihydroxyacetone‐phosphate, were found to be increased in the RFX6‐KO group (Figure S4H). Consistent with the reduced lactate, these increased intermediates suggested deceleration and blockage of glycolytic flux induced by silencing RFX6. Further, a 2‐PG production assay verified that silencing RFX6 reduced the 2‐PG production while overexpressing RFX6 augmented its production in vitro (Figures 5J,K and S6L,M). In the retrospective analysis of the metabolome profiling data from tumour tissues, levels of 2‐PG, phosphoenolpyruvate and pyruvate, which were downstream products of PGAM1, were observed to decrease in the RFX6‐KO group and increase in the RFX6‐OE group (Figure S5A,B). These results demonstrate that RFX6 transcriptionally upregulates the expression of PGAM1 and its enzymatic activity.

### 3.6 RFX6 promotes HCC progression via a PGAM1‐dependent pathway

**FIGURE 6:**
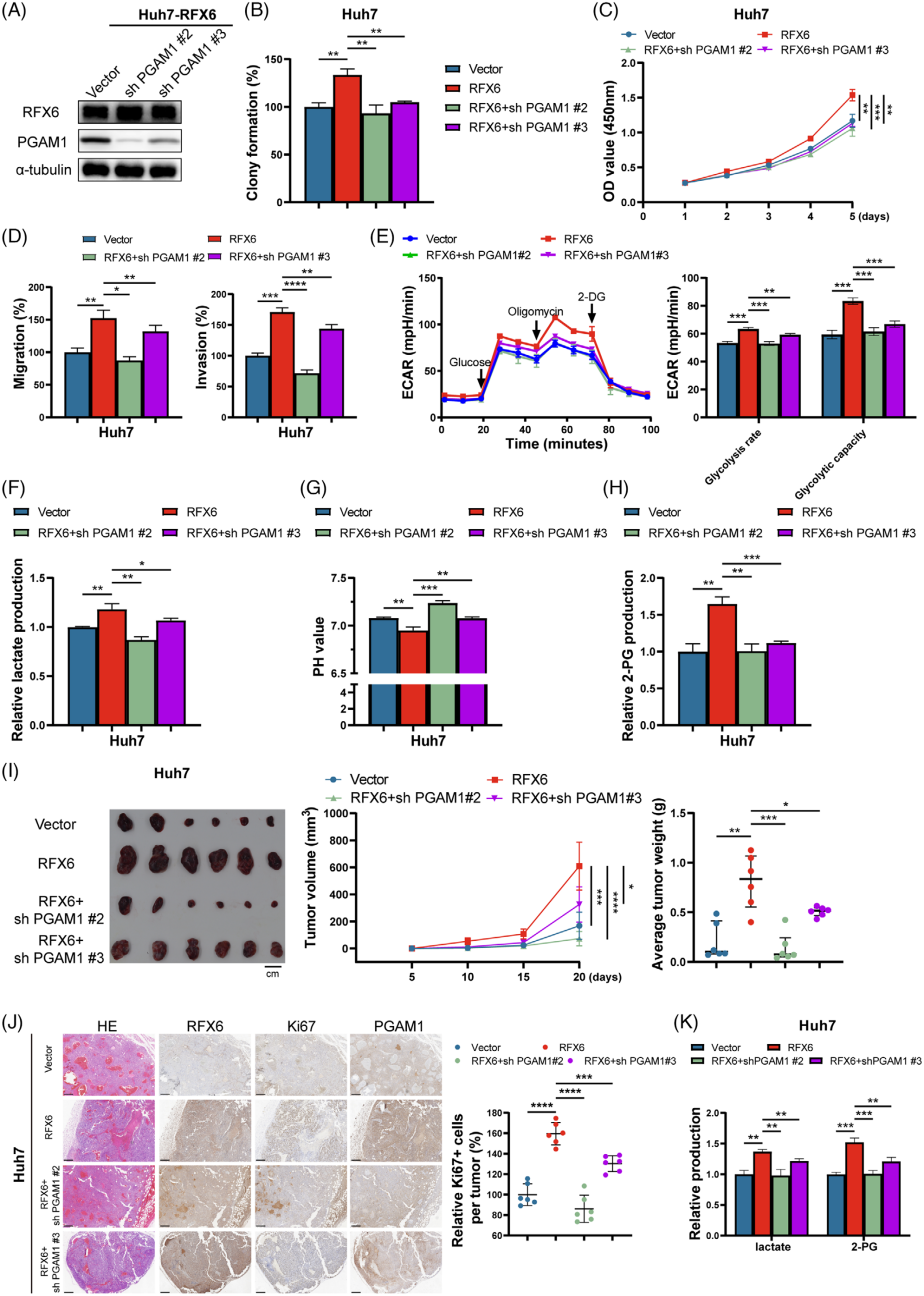
Regulatory factor X6 (RFX6) promotes hepatocellular carcinoma (HCC) progression via a phosphoglycerate mutase 1 (PGAM1)‐dependent pathway. (A) Western blotting demonstrates the efficiency of stable PGAM1‐knockdown (KD) in RFX6‐overexpressing (OE) Huh7 cells. (B) Stable PGAM1‐KD attenuated the RFX6‐induced proliferation of HCC cells, as shown by the colony formation assay. (C) Stable PGAM1‐KD attenuated the RFX6‐induced proliferation of HCC cells, as shown by the Cell Counting Kit‐8 (CCK‐8) assay. (D) Stable PGAM1‐KD attenuated the RFX6‐induced motility of HCC cells, as shown by the transwell assay. A total of 1.6 × 10^5^ cells were seeded in a chamber, and incubated for 12 h (migration assay) or 36 h (invasion assay). (E–H) Stable PGAM1‐KD attenuated the RFX6‐induced glycolysis in HCC cells, as determined by Seahorse experiments (E), lactate production assay (F), pH measurements (G) and 2‐phosphoglycerate (2‐PG) production assay (H). (I) Stable PGAM1‐KD attenuated the RFX6‐induced HCC growth, as indicated by tumour volumes (left and middle panel) and weights (right panel) (*n* = 6/group). Scale bar, 1 cm. (J) Stable PGAM1‐KD attenuated the RFX6‐induced HCC growth, as indicated by the quantitation of Ki67+ stained cells. Scale bar, 250 μm. (K) Stable PGAM1‐KD attenuated the RFX6‐induced glycolysis in HCC cells in vivo, as shown by lactate and 2‐PG production assays. Data are represented as mean ± standard deviation (SD) from at least three independent experiments. A two‐tailed Student's *t*‐test was used for statistical analysis. ^*^ *p* < .05, ^**^ *p* < .01, ^***^ *p* < .001 and ^****^ *p* < .0001.

To determine whether RFX6 promoted HCC progression via a PGAM1‐dependent pathway, rescue experiments were performed in PGAM1‐KD cells (Figures 6A and S7A,B). As demonstrated by functional assays, cell proliferation and motility induced by RFX6 were reversed by silencing PGAM1 (Figures 6B–D and S7C–I). Similarly, experiments measuring ECAR, lactate production, 2‐PG production, and pH levels demonstrated that RFX6‐enhanced glycolysis was reversed by silencing PGAM1 (Figures 6E–H and S7J,K). Subcutaneous models revealed that silencing PGAM1 suppressed the RFX6‐induced cell proliferation in vivo, presenting as attenuated tumour volumes, tumour weights and Ki67+ cells in the stable PGAM1‐KD groups (Figure 6I,J). Moreover, metabolite levels showed that silencing PGAM1 markedly suppressed the RFX6‐induced glycolysis in vivo, presenting as attenuated lactate and 2‐PG production in the subcutaneous tumours from the stable PGAM1‐KD groups (Figure 6K).

Additionally, PGAM1‐OE plasmids were transduced into RFX6‐KO cells, and related rescue experiments were carried out (Figure S8A). As shown by functional assays, overexpressing PGAM1 reversed the suppression of cell proliferation and motility by silencing RFX6 (Figure S8B–E). Lactate production, 2‐PG production and pH measurements showed that overexpressing PGAM1 released the inhibition of glycolysis induced by RFX6‐KO (Figure S8F,G). Supplementation with 2‐PG partly rescued the suppression of cell proliferation caused by silencing RFX6 (Figure S6N). Overall, these in vitro and in vivo results demonstrate that RFX6 facilitates glycolysis, growth and metastasis of HCC through transcriptional regulation of PGAM1.

## 4 DISCUSSION

**FIGURE 7:**
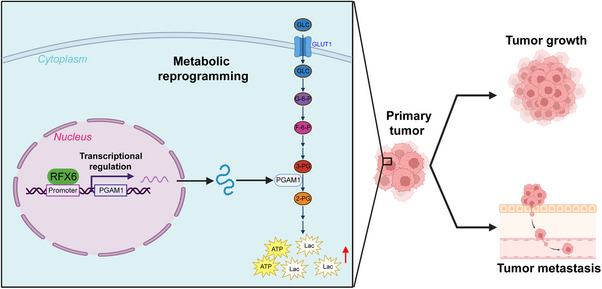
Schematic diagram of the underlying mechanisms of regulatory factor X6 (RFX6) promotion on hepatocellular carcinoma (HCC) progression via the phosphoglycerate mutase 1 (PGAM1)‐dependent glucose metabolism.

Previous studies have highlighted the linkage between the aberrant RFX6 expression and cancer development.^12^, ^13^, ^14^, ^15^, ^16^ However, the precise role of RFX6 in HCC remained ambiguous. In the current study, RFX6 was significantly increased in HCC. Upregulated RFX6 represented an unfavorable prognostic factor in HCC patients. Further, RFX6 exerted a tumour‐promoting effect on HCC growth and metastasis. By transcriptionally upregulating PGAM1, RFX6‐enhanced glycolysis, thereby promoting HCC progression (Figure 7). Our results indicated that RFX6 might serve as a potential biomarker for predicting recurrence or metastasis, as well as a feasible therapeutic target.

Aerobic glycolysis serves as a critical hallmark of HCC, but its intrinsic mechanisms are still unknown. Based on our ChIP‐seq and metabolome profiling, KEGG analysis found that central carbon metabolism in cancer was one of the pathways potentially regulated by RFX6. Further experiments associated with glucose metabolism identified the promoting effect of RFX6 on glycolysis in HCC. Moreover, PGAM1, a key enzyme of glycolysis was recognised as one of the downstream targets of RFX6 in the present study. PGAM1 was shown to exert pro‐tumoural functions through its metabolic activity.^19^, ^20^, ^21^, ^22^, ^23^, ^24^ By rescue experiments, PGAM1 was found to be critically responsible for RFX6‐mediated glycolysis, growth and metastasis of HCC. To the best of our knowledge, the current study provided the first evidence delineating the link between RFX6 and glycolysis in HCC.

A previous study showed that RFX6 might contributes to the HCC progression through the Notch pathway.^14^ Concurrently, this study also exhibited that RFX6 expression was inversely correlated with the dysfunction of progressive T cell, suggesting its functions in reshaping tumour microenvironment. Although the related pathways did not exhibit significance in the KEGG analyses of the current study, potentially attributed to discrepancies between cell lines and experimental methods, a genome‐scale CRISPR‐Cas9 screen conducted by Pan et al. identified *RFX6* as one of the genes that sensitised tumour cells to T‐cell‐mediated killing.^34^ Moreover, PGAM1 was recently reported to inhibit T‐cell infiltration via ferroptosis suppression in HCC.^35^ These evidences, along with our study, indicate the instrumental role of RFX6/PGAM1 axis in the development of HCC.

As mentioned above, glycolytic enzymes might exert diverse pro‐tumoural functions, facilitating tumour progression in a synergistic manner.^7^, ^36^, ^37^, ^38^ A recent study recognised the assembly of penultimate enzyme complexes, including PGAM1, in breast cancer cells. Through rapid channeling of glycolytic intermediates, this enzyme complex accelerates glucose uptake and augments glycolysis.^39^ An elevated glucose consumption was observed in the RFX6‐OE cells. It was of interest to postulate that RFX6 might mediate the assembly of such complexes via the regulation of PGAM1 in HCC cells, resulting in an increased glycolytic flux under Warburg effect conditions.

In addition to critical involvement in glycolysis, PGAM1 participates in two other biosynthetic pathways, pentose phosphate and serine biosynthesis pathway.^40^ Suppression of PGAM1 results in the accumulation of 3‐PG, which subsequently inactivates 6‐phosphogluconate dehydrogenase involved in the pentose phosphate pathway, consequently impairing de novo nucleotide biosynthesis. Deprivation of 2‐PG by PGAM1 inhibition also remarkably attenuates the activity of phosphoglycerate dehydrogenase, which utilises 3‐PG to initiate the serine biosynthesis pathway. These observations collectively suggest that targeting the RFX6–PGAM1 axis might be a tempting option for the treatment of HCC, not only by restricting energy supply but also by impeding anabolic processes essential for tumour cell proliferation, thereby ‘killing two birds with one stone’.^41^

In prostate cancer, the risk‐associated T allele increases the binding of homeobox B13 (HOXB13) and androgen receptor (AR), supporting allele‐specific upregulation of RFX6.^13^ Similarly, HCC tissues exhibit elevated expression of HOXB13 and AR compared to normal tissues, implying their tumour‐associated properties and clinical relevance.^42^, ^43^, ^44^, ^45^ Whether upregulated HOXB13 and AR would enhance the expression of RFX6, thereby triggering a transcription factor cascade in the development of HCC, remains to be explored. Moreover, the failure of clinical trials examining the efficacy of AR antagonists suggests its double‐edged role in HCC.^46^, ^47^ Therefore, targeting the pro‐tumoural downstream of AR (i.e., RFX6), might be a more efficient strategy.

In line with Warburg's theory, central carbon metabolism in cancer was characterised by enhanced glycolysis and suppressed oxidative phosphorylation (OXPHOS) in tumour cells.^48^ Although OXPHOS is the preferred energy production process in HCC stem cells, the switch to the glycolysis takes place during cell differentiation, accompanied by epigenetic alterations.^49^ Therefore, RFX6 might transcriptionally regulate genes involved in OXPHOS, reprogramming glucose metabolism in HCC.

Aerobic glycolysis in tumour cells produces a large amount of lactate, which induces tumourigenesis by provoking acidosis, angiogenesis and immunosuppression.^50^ A recent study by Zhang et al. reported a new post‐translational modification, histone lactylation, where lactate can modify lysine residues of histones.^51^ In ocular melanoma, histone lactylation promotes tumour progression by regulating the transcription of an m^6^A reader protein.^52^ Given the excessive lactate generated by RFX6 regulation, it can be hypothesised that lactate modifies histones associated with the promoter region of RFX6, forming a positive feedback loop. However, whether this novel epigenetic mechanism participates in the HCC tumourigenesis remains to be explored.

In conclusion, our study recognised *RFX6* as an essential gene for tumour development and uncovered its effect on HCC progression. We first revealed the impact of RFX6 on glycolysis and further demonstrated the significance of this novel RFX6–PGAM1 regulatory axis in HCC progression. Our study might provide a novel therapeutic target in HCC.

## AUTHOR CONTRIBUTIONS

*Conceptualisation*: Binkui Li, Yunfei Yuan and Yi Niu. *Methodology*: Zhiyu Qiu, Chenwei Wang and Pinzhu Huang. *Investigation*: Yichuan Yuan and Yunxing Shi. *Visualisation*: Zhu Lin and Zhenkun Huang. *Statistical analysis*: Zhu Lin and Dinglan Zuo. *Supervision*: Binkui Li, Yunfei Yuan, Yi Niu, Wei He and Jiliang Qiu. *Writing—original draft*: Zhiyu Qiu, Chenwei Wang and Pinzhu Huang. *Writing—review and editing*: Binkui Li, Yunfei Yuan, and Yi Niu.

## CONFLICT OF INTEREST STATEMENT

The authors declare they have no conflicts of interest.

## ETHICS STATEMENT

This study complied with the standards of the 1975 Declaration of Helsinki and the experiments were approved by the Ethics Committee of SYSUCC. Written informed consent was obtained from patients who provided samples. The animal experiments were approved by the institutional ethics committee of Sun Yat‐Sen University, Guangzhou, China.

## Supporting information

Supporting Information

Supporting Information

Supporting Information

Supporting Information

Supporting Information

Supporting Information

Supporting Information

Supporting Information

Supporting Information

Supporting Information

Supporting Information

Supporting Information

Supporting Information

Supporting Information

Supporting Information

Supporting Information

## Data Availability

The ChIP‐seq and RNA‐seq data generated in this study have been deposited in the GEO database under the accession numbers GSE248062 and GSE248063. The remaining data are available within main text or supplementary materials or source data files. Source data are provided with this paper.
