# Supplementary figures and images for: RFX6 facilitates aerobic glycolysis‐mediated growth and metastasis of hepatocellular carcinoma through targeting PGAM1
Source: Clin Transl Med. 2023 Dec 13;13(12):e1511. doi: 10.1002/ctm2.1511 (PMC10719540; doi:10.1002/ctm2.1511)

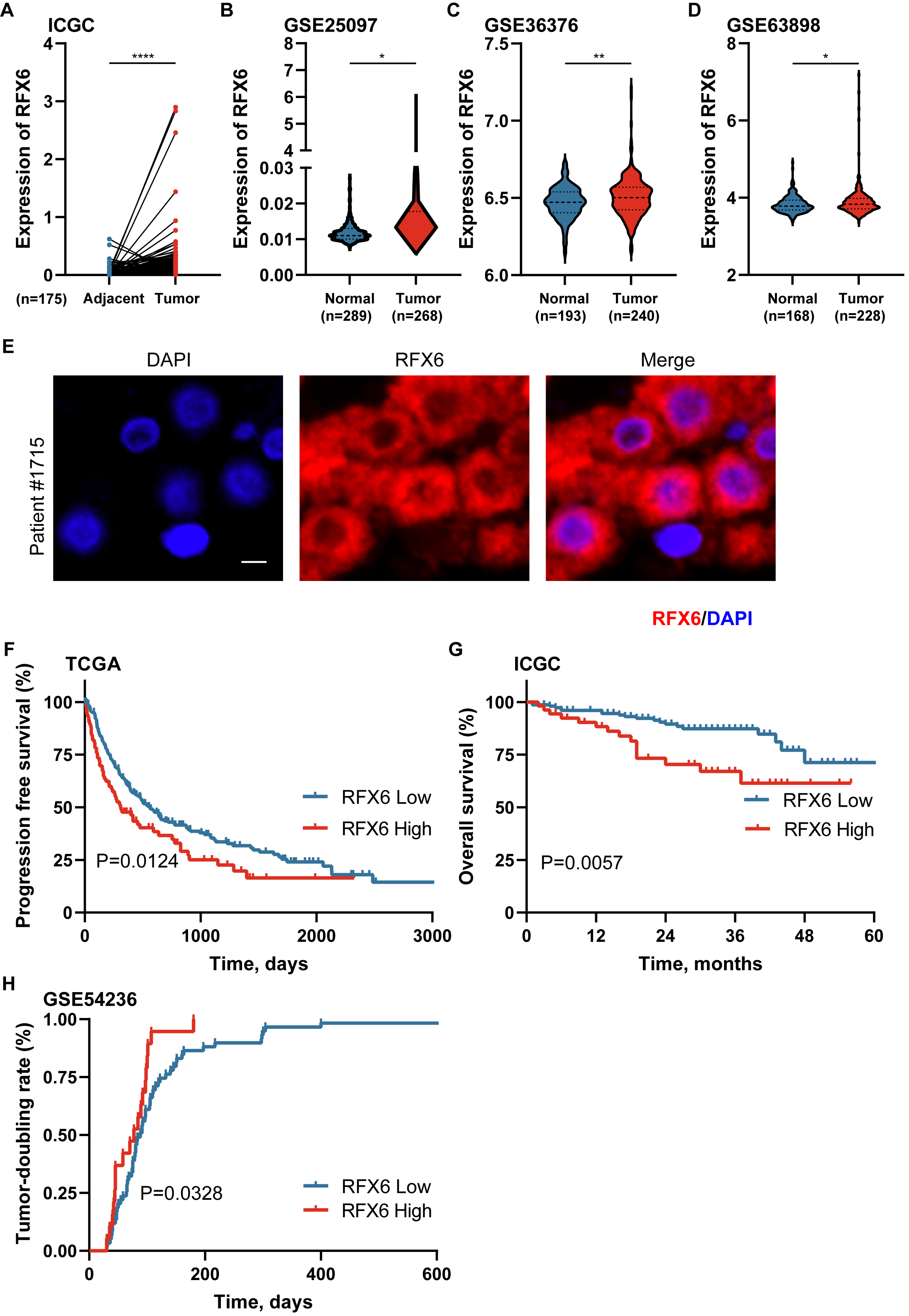

Supplement: Supplementary file 1 — Supporting Information [file CTM2-13-e1511-s012.tif]

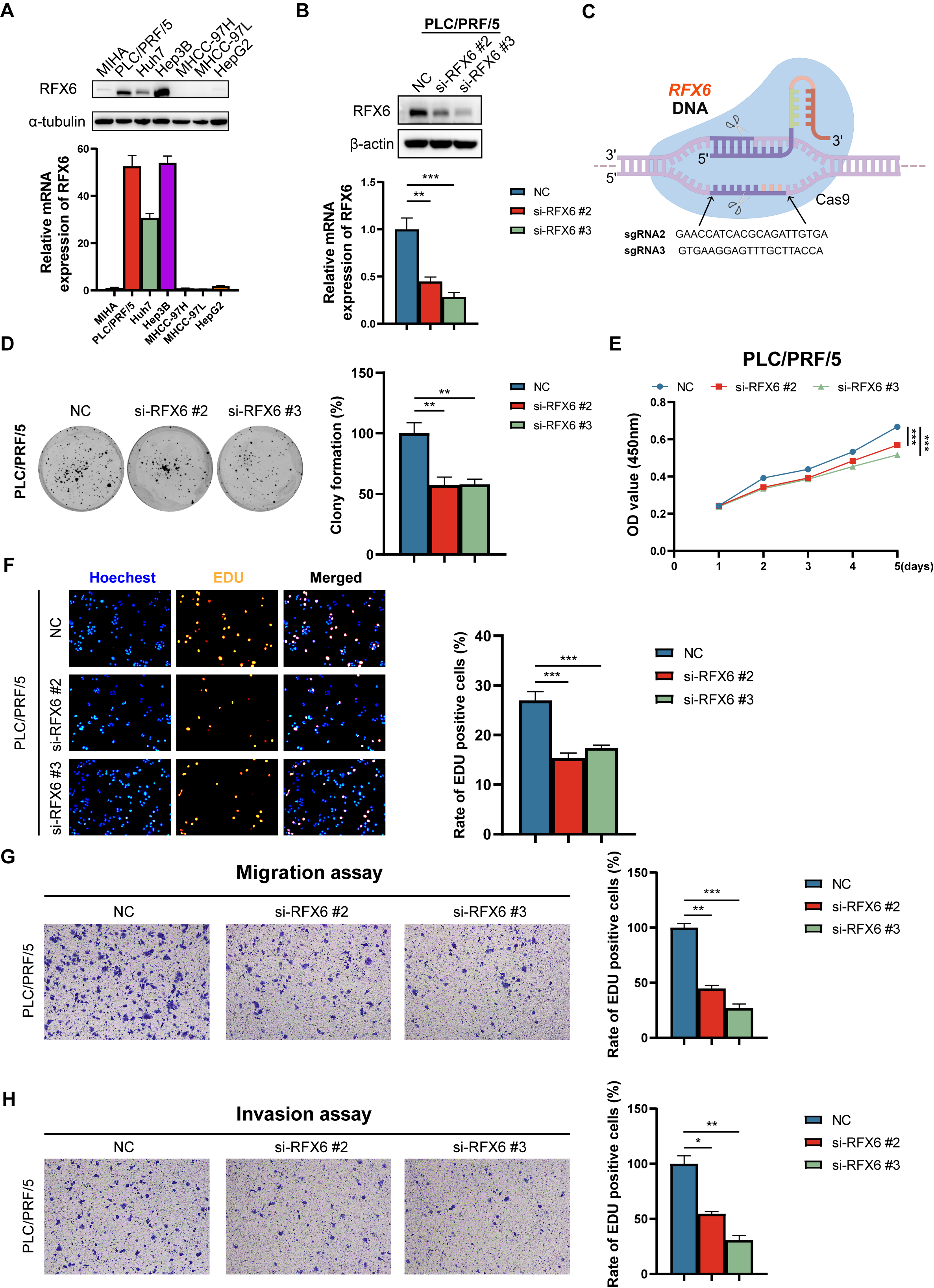

Supplement: Supplementary file 2 — Supporting Information [file CTM2-13-e1511-s010.tif]

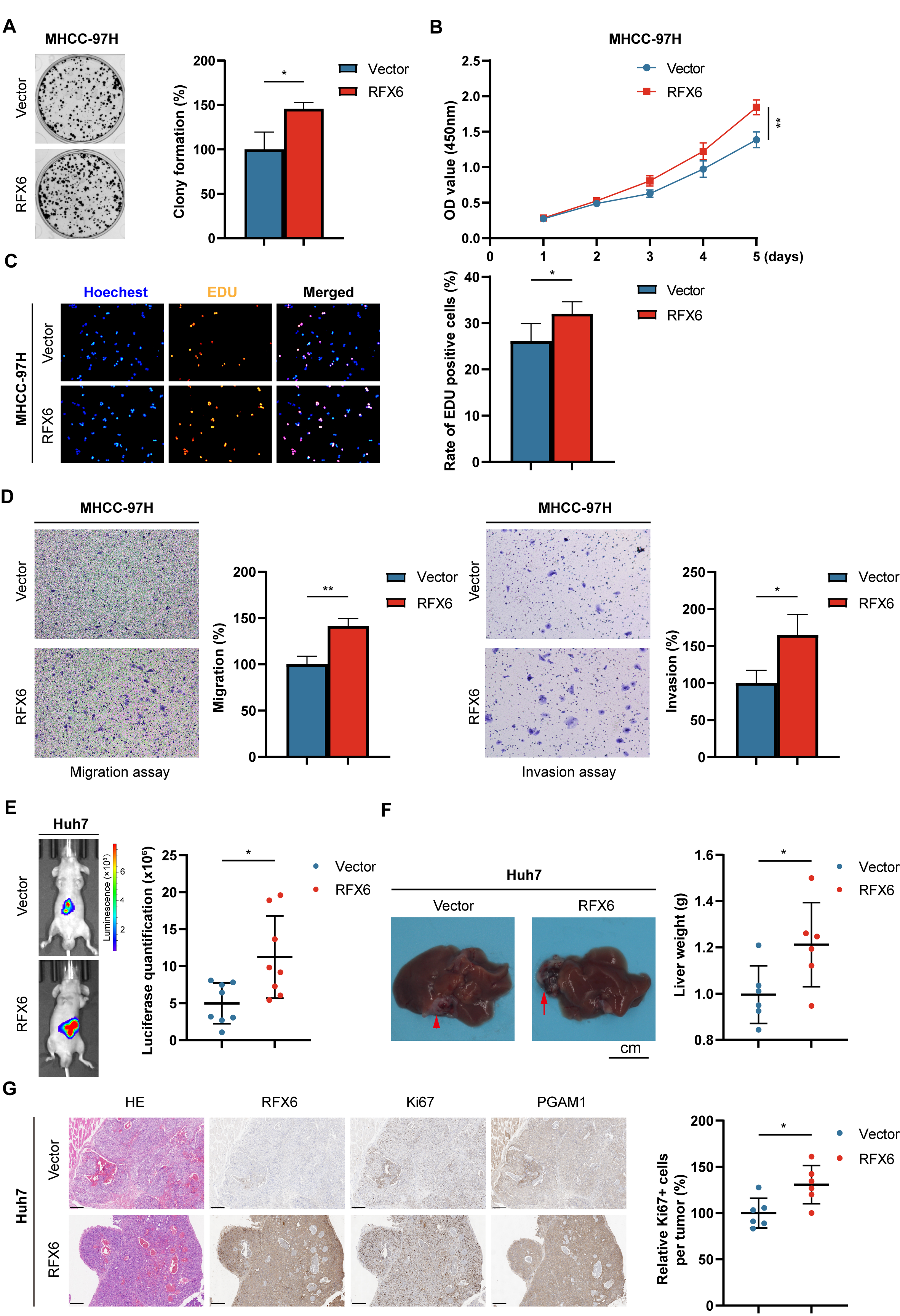

Supplement: Supplementary file 3 — Supporting Information [file CTM2-13-e1511-s004.tif]

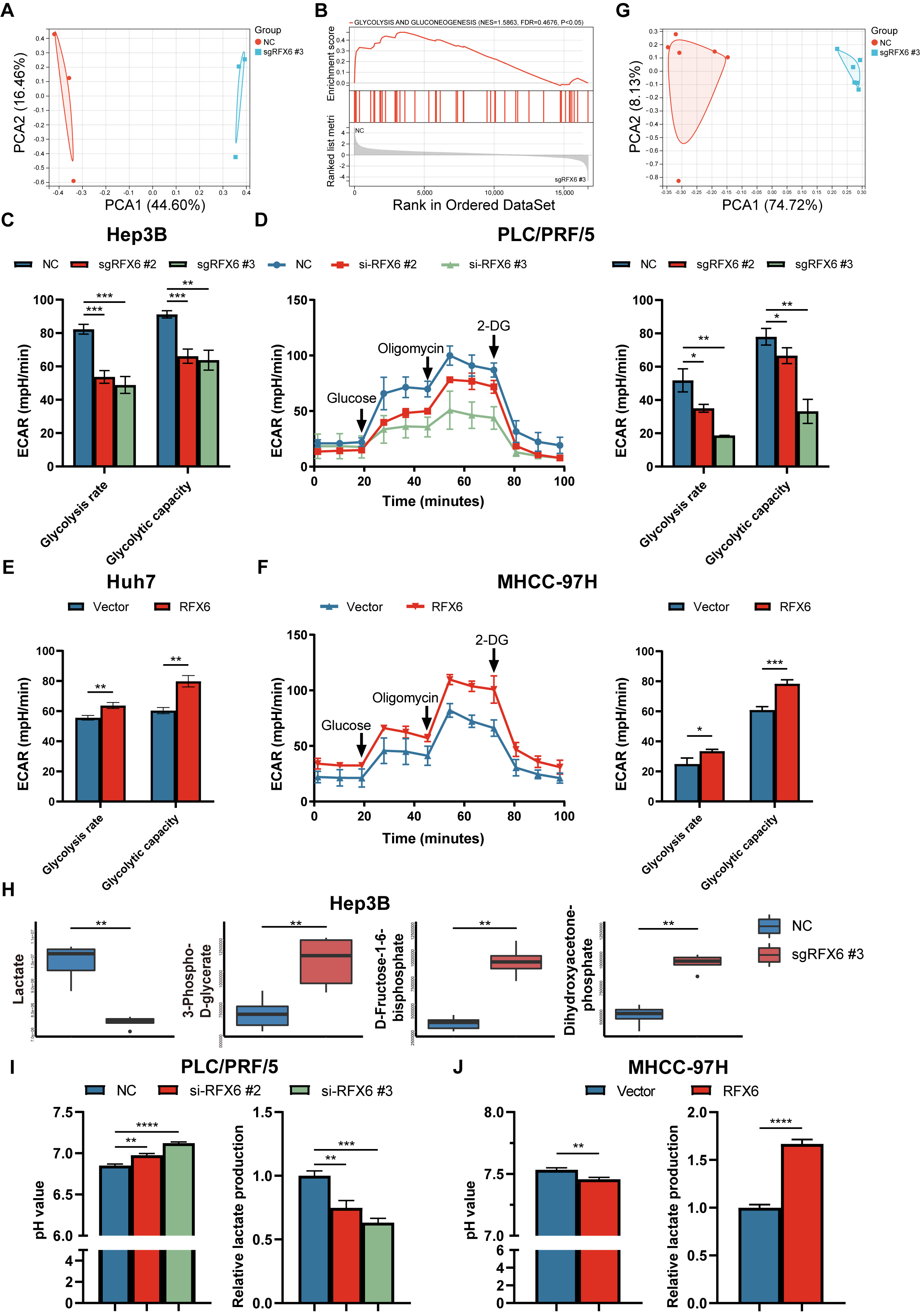

Supplement: Supplementary file 4 — Supporting Information [file CTM2-13-e1511-s008.tif]

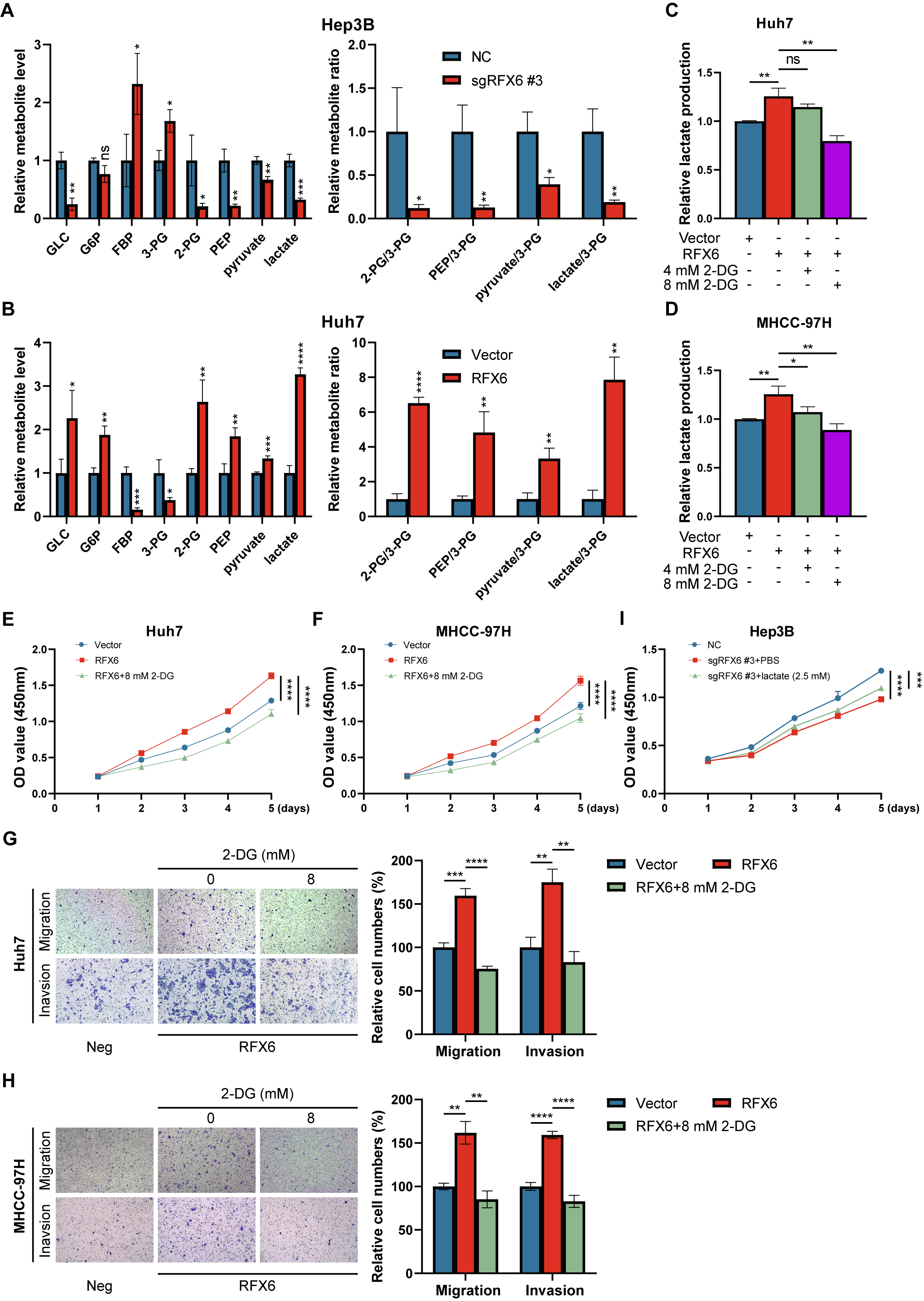

Supplement: Supplementary file 5 — Supporting Information [file CTM2-13-e1511-s001.tif]

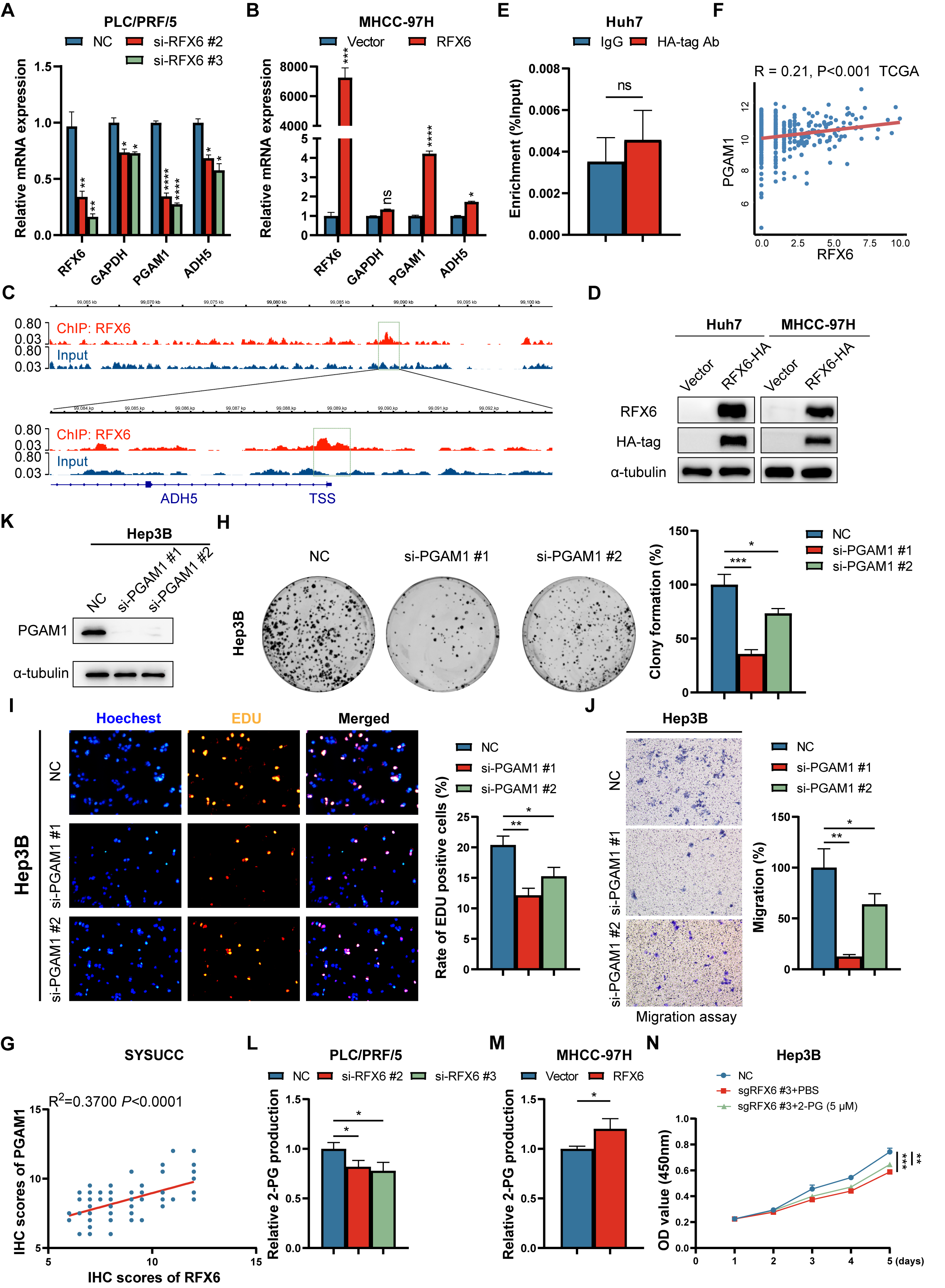

Supplement: Supplementary file 6 — Supporting Information [file CTM2-13-e1511-s011.tif]

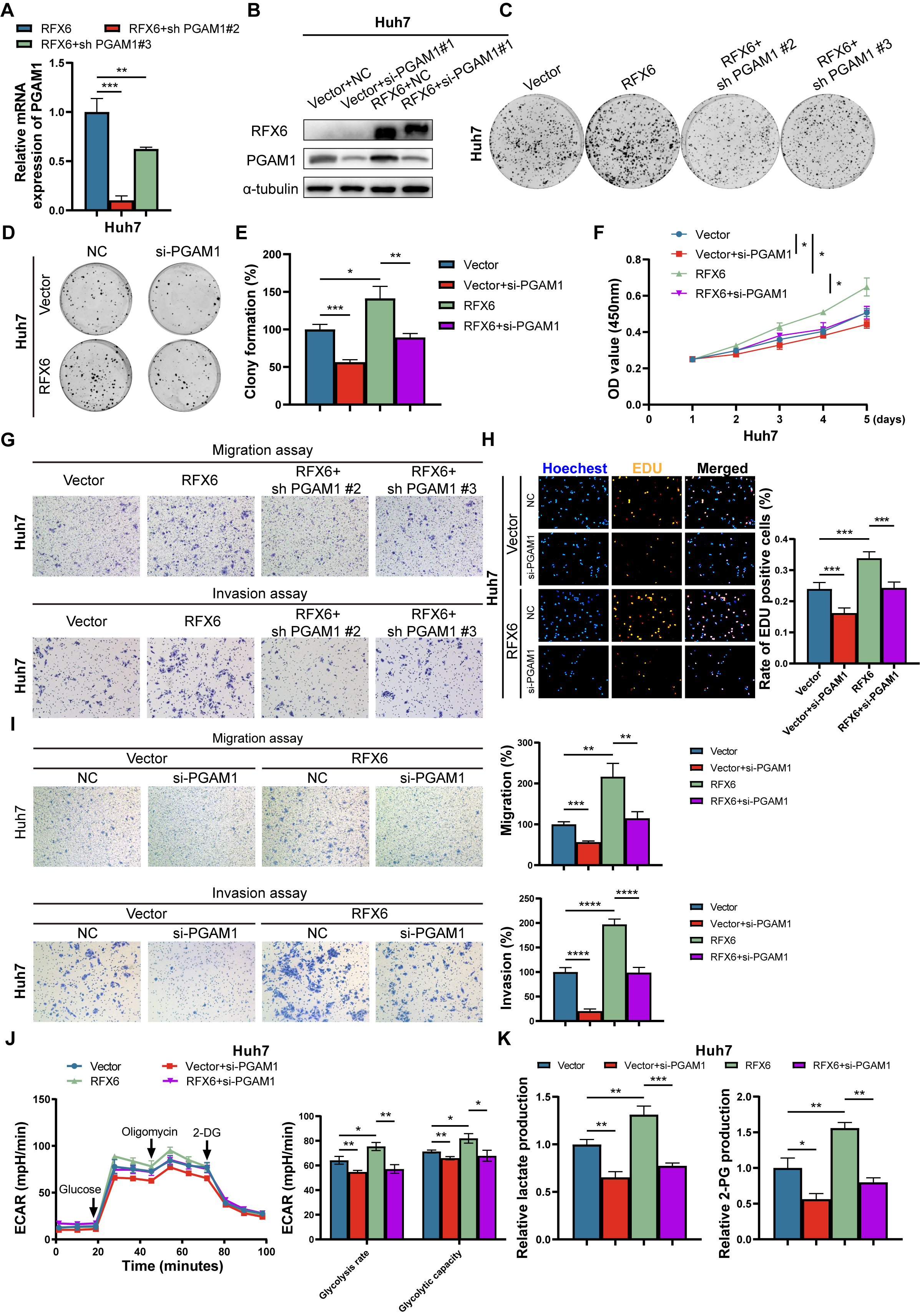

Supplement: Supplementary file 7 — Supporting Information [file CTM2-13-e1511-s014.tif]

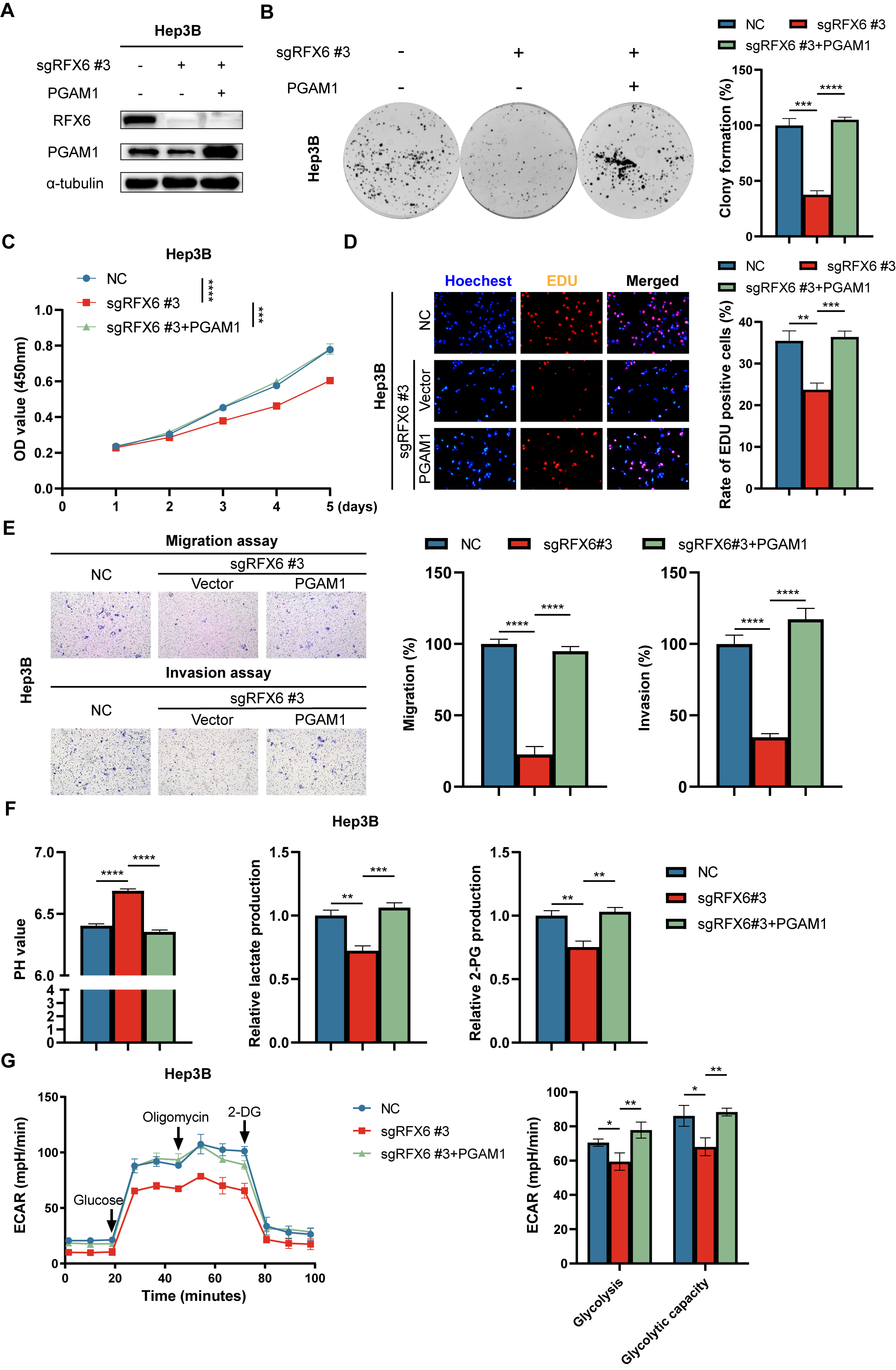

Supplement: Supplementary file 8 — Supporting Information [file CTM2-13-e1511-s006.tif]
