# Supplementary material for: RFX6 facilitates aerobic glycolysis‐mediated growth and metastasis of hepatocellular carcinoma through targeting PGAM1
Source: Clin Transl Med. 2023 Dec 13;13(12):e1511. doi: 10.1002/ctm2.1511 (PMC10719540; doi:10.1002/ctm2.1511)
Supplement: Supplementary file 9 — Supporting Information [file CTM2-13-e1511-s009.docx]

**RFX6 facilitates aerobic glycolysis-mediated growth and metastasis of hepatocellular carcinoma through targeting PGAM1**

Zhiyu Qiu^1,2^, Chenwei Wang^1,2^, Pinzhu Huang^3^, Yichuan Yuan^1,2^, Yunxing Shi^1,2^, Zhu lin^1,2^, Zhenkun Huang^1,2^, Dinglan Zuo^1^, Jiliang Qiu^1,2^, Wei He^1,2^, Jingxian Shen^1,4^, Yi Niu^1^, Yunfei Yuan^1,2^, Binkui Li^1,2^

**Table of contents**

Supplementary materials and methods

Supplementary figure legends

**Materials and Methods**

**Analysis of public clinical datasets**

RNA sequencing and clinical data of tissues were downloaded from The Cancer Genome Atlas (TCGA) database (https://portal.gdc.cancer.gov), International Cancer Genome Consortium (ICGC) database (https://dcc.icgc.org/), and National Center for Biotechnology Information Gene Expression Omnibus (GEO) database (https://www.ncbi.nlm.nih.gov/geo/). Comparison of RFX6 expression in paired tumor and normal tissues samples as well as survival analyses between groups with different RFX6 expression were performed in the R programming language (R version 4.1.2; R Foundation for Statistical Computing, Vienna, Austria). In different datasets, OS and progression free survival (PFS) were defined according to related instructions.

**Cell lines and cell culture**

Human HCC cell lines (PLC/PRF/5, Huh7, Hep3B, MHCC-97H, MHCC-97L, and HepG2), MIHA (the normal liver cell line), and HEK293T cell line were purchased from the Shanghai Cell Bank of the Chinese Academy of Sciences (Shanghai, China) with short tandem repeat appraisal certificates. As described in previous publications, cells were maintained in Dulbecco’s Modified Eagle medium (ThermoFisher, Massachusetts, USA) supplemented with 10% fetal bovine serum (Gibco, California, USA) at 37 °C in 5% CO2.

**RFX6 knockout by CRISPR/Cas9**

sgRNA design and cloning was performed according to the Feng Zhang lab general cloning protocols. RFX6 sgRNAs oligos were designed based on the target site sequence and are flanked on the 3′ end by a 3 bp NGG PAM sequence. With the use of the Cas9 target design tools (http://www.genome-engineering.org), three sgRNAs were designed for each target:

RFX6 sg1 forward: 5′-GAAATTAGAGCCAGCCTGTG-3′;

RFX6 sg1 reverse: 5′-CACAGGCTGGCTCTAATTTC-3′;

RFX6 sg2 forward: 5′-GAACCATCACGCAGATTGTGA-3′;

RFX6 sg2 reverse: 5′-TCACAATCTGCGTGATGGTTC-3′;

RFX6 sg3 forward: 5′-GTGAAGGAGTTTGCTTACCA-3′;

RFX6 sg3 reverse: 5′-TGGTAAGCAAACTCCTTCAC-3′;

Control sgRNA forward: 5′- GACCGGGGCGAGGAGCTGTTCACCG -3′;

Control sgRNA reverse: 5′- CGGTGAACAGCTCCTCGCCCCGGTC -3′;

The sgRNAs were cloned into the lentiCRISPRv2 vector (Addgene). For lentivirus production, cloned lentiCRISPRv2 plasmids were co-transfected into HEK293T cells with the packaging plasmids pVSVg (AddGene 8454) and psPAX2 (AddGene 12260). GeneCopoeia™ Lentivirus were harvested and Hep3B cells were infected with two sgRNA dependently for RFX6. 48 h after infection, cells were placed under puromycin selection for one week and single-cell-derived clones were picked and expanded. Finally, the efficiency of RFX6-knockout was verified by quantitative real-time PCR (qRT-PCR) and western blotting.

**Lentiviral transduction and generation of stable cell lines**

Lentiviruses were produced by transfecting HEK293T cells with short hairpin RNA (shRNA)-targeting plasmids and the helper plasmids psi-LVRU6GP. The cell supernatants were harvested 48 h after transfection and were either used to infect cells or stored at −80 °C. To obtain stable cell lines, cells were infected at low confluence (20%) for 12 h with lentiviral supernatants diluted 1:1 with normal culture medium in the presence of 5 ng/ml of polybrene (Sigma; Missouri, USA). 48 h after infection, cells were placed under Blasticidin S (ThermoFisher, Massachusetts, USA) selection for five days and then passaged before use. Blasticidin S was used at 2 μg/ml to maintain Huh7 cells. Typical four shRNAs were purchased from GeneCopoeia (Rockville, Maryland, USA), and the most effective two shRNAs were used for the experiments. qRT-PCR and western blotting were performed to verify the efficiency of PGAM1 overexpressed or depletion expression.

**RNA interfering**

Small interfering RNA for RFX6 and PGAM1 were purchased from Genepharma (Shanghai, China) and RIBOBIO (Guangzhou, China). Reverse transfection of small interfering RNA was performed with Lipofectamine-RNAiMAX (Invitrogen, Califonia, USA). After 24 h, the supernatant was replaced with fresh medium and the down-regulation efficiency was verified by qRT-PCR and western blotting 48 h after co-transfections. The targeting sequences of sgRNAs were used to construct small interfering RNAs. The targets of PGAM1 small interfering RNA sequences were:

siPGAM1#1, 5’-CCAGTGGAAGACGAATGT A-3’

siPGAM1#2, 5’-AGGTTCTCAGTCTAAGCT A-3’.

**Immunohistochemical (IHC) staining**

After deparaffinized and blocked off the nonspecific antigen with goat serum (Zsbio, Beijing, China), HCC tissue microarrays and sections were probed with primary antibodies, as described in Table S10, overnight at 4 °C. Then, the microarrays and sections were incubated with HRP anti-rabbit/mouse antibodies (Dako, Copenhagen, Denmark) for half an hour at 37 °C, and then the diaminobenzidine chromogen (Dako, Copenhagen, Denmark) was applied for reaction, followed by counterstaining with hematoxylin (Leagene, Beijing, China).

**Tyramide signal amplification (TSA)**

Uniplex immunofluorescent (IF) staining was performed manually by using individual TSA-conjugated fluorophores to detect various targets within an IF assay. After deparaffnization, slides were placed in a plastic container filled with 100 mL of antigen retrieval (AR) solution (citric acid solution, pH6.0/pH9.0); microwave technology was used to bring the liquid to the boiling point (1 min) with 100% power, and the slides were then microwaved for an additional 15 min with 20% power. The slides were allowed to cool in the AR buffer for 15 min at room temperature and were then washed with distilled water and 1×Tris-buffered saline with Tween 20 (TBST; Santa Cruz Biotechnology, Texas, USA) in a separate slide container. Then the slides were covered with blocking solution and incubated for 10 min. In situ hybridization chamber with moisturized paper towel were used on the bottom. Primary antibodies were added (100 μL/ tissue) and then the slides were incubated for 1 hat room temperature. Next, the slides were washed with TBST for 3 min × 2 times and incubated for 10 min at room temperature with anti-mouse or anti-rabbit secondary antibodies.

Next, the slides were washed with TBST for 3 min × 2 times and then incubated at room temperature for 10 min with PPD-520 DYE (1:100 dilution, 100 μL/tissue). After three additional washes in TBST, the slides were counterstained with DAPI (1:2000 dilution) for 5 min and mounted with Olympus VS200 MTL (Olympus, Hamburg, Germany).

**Chromatin immunoprecipitation (ChIP) assay**

ChIP assays were performed in both MHCC-97H (using ChIP-semiquantitative PCR) and Huh7 (using ChIP-qPCR) cells with a ChIP kit (Cell Signaling Technology, Massachusetts, USA) according to the manufacturer’s instruments.

Briefly, 1% formaldehyde (Sigma-Aldrich, Germany) solution was added to induce MHCC-97H or Huh7 cell crosslinking followed by glycine solution to quench the reaction. Afterwards, the cells were lysed, and the nucleoprotein complexes were sonicated for 10 cycles of 10 s power-on and 20 s interval with an intensity of 200 W with the sonicate conductor (Qsonica, Connecticut, USA). Then, anti-HA antibody or IgG, as described in Table S10, was added and incubated with the complexes overnight at 4 °C. The next day, Protein A/G magnetic beads were added to precipitate the indicated fragments for an addition 4 h at 4 °C.

For ChIP-semiquantitative PCR, after extraction and purification of the indicated DNA, PCR and electrophoresis were performed to identify PGAM1/ADH5.

For ChIP-qPCR, after extraction and purification of the indicated DNA, samples were analyzed in triplicate and the amount of immunoprecipitated DNA was normalized to the input. The PCR primers were present in Table S9.

**ChIP-seq**

ChIP-seq was performed in MHCC-97H cells with a ChIP kit (Cell Signaling Technology, Massachusetts, USA) according to the manufacturer’s instruments. ChIP-seq analysis was conducted by Novogene Co., Ltd (Beijing, China). Libraries were sequenced on an Illumina novaseq6000 platform. MACS version 2.1.0.2 software was run with the mapped reads to detect the statistically significant ChIP-enriched peaks with a significant cut-off q value threshold of 0.01. Gene visualization was carried out by Integrative Genomics Viewer.

**Luciferase reporter assay**

Luciferase reporter plasmids, including full-length and mutant PGAM1 promoters, were constructed by Kidan Bio Co. Ltd. (Guangzhou, China). For this assay, Hep3B, PLC/PRF-5, Huh7, and MHCC-97H cells (5×10^4^) were seeded in 24-well plates and transfected with the indicated plasmids using Lipofectamine™ 3000 (Invitrogen, Califonia, USA) according to the manufacturer’s instructions. After 48 h, the conditioned medium was collected, and the Gaussia luciferase (Gluc) and Renilla luciferase (Rluc) activities were measured with a luminometer (PerkinElmer, Massachusetts, USA) consecutively using the Luc-Pair™ Duo-Luciferase HS Assay Kit (GeneCopoeia, Rockville, Maryland, USA) according to the manufacturer’s instructions. The Gluc activity was normalized to Rluc activity. Each group was performed in triplicate, and the data represent the mean ± Standard deviation of three independent experiments.

**RNA extraction, qRT-PCR, and primers**

Total RNAs were prepared using the RNA-Quick Purification kit (ESscience Biotech) according to the manufacturer’s instructions. A total of 1 μg of RNA was reverse-transcribed using cDNA synthesis with random hexamers and Superscript III (Invitrogen, Califonia, USA). The cDNA templates were subjected to PCR amplification. Then, qRT-PCR was conducted with the use of SYBR Green PCR kit (Invitrogen, Califonia, USA) on LightCycle 480 II detector (Roche, Basel, Switzerland). All experiments were run in triplicate and target gene-expression levels were normalized to ACTB as control. DNA primer sequences that were used to detect target gene expression by qRT-PCR are listed in supplementary Table S9.

**Western blotting analysis, antibodies and chemicals**

In brief, cells were lysed by RIPA buffer (P0013B, Beyotime, China) containing the complete cocktail of protease inhibitors (#11836153001, Roche, Switzerland). Protein concentrations were determined with the BCA protein assay kit (P0011, Beyotime, China). Proteins were separated by 10% SDS-PAGE and transferred to nitrocellulose filters, and blotted with related primary antibodies at 4 °C overnight. Then the proteins were incubated with secondary antibodies in room temperature for 1 h. The nitrocellulose filters with target protein were exposed in visualizer (4600, Tanon, China). Western blotting analysis was performed with specific antibodies and secondary anti-mouse or anti-rabbit antibodies conjugated to horseradish peroxidase (Amersham Biosciences). Visualization was achieved by chemiluminescence (Bio-rad). The antibodies used for Western blotting, IHC, and ChIP were listed in supplementary Table S10.

**In vitro transwell migration, invasion assays**

Transwell chambers (8 μm por size; Costar) and chambers with Matrigel (Kennebunk, Maine, USA) were used to assess in vitro cell migration and invasion, respectively according to the manufacturer’s instructions. HCC cells were seeded into the top chamber of each insert (1.6-3.2×10^5^ cells in 200 μL serum-free DMEM per well), at the same time, DMEM (800 μL, containing 10% FBS) was added to the bottom chamber. After a 12-36h incubation at 37 °C, the cells on the bottom surface of transwells were fixed in methanol, stained with 0.1% crystal violet, and counted under the microscope.

**Cell proliferation and colony formation assays**

Cell Counting kit-8 (CCK-8; DongDo, Japan) was used to assess cell proliferation. Firstly, HCC cells were seeded into 96-well plates and cultured. The relative cell density was measured by Biotek Epoch 2 machine (BioTek, Winooski, Vermont, USA) at 450 nm 2 h after adding CCK-8 mixtures (fresh medium: CCK-8 reagent = 10:1). Five repeated wells were used for each experimental condition, and three independent experiments were performed.

For EDU cell proliferation assay, cells were seeded in a 24-well plate with corresponding concentration of EDU reagent for 2.5 h. Cells were washed with PBS for 3 min twice before incubating with 4% Paraformaldehyde for 30 min, and permeated with 0.3% TritonX-100 in PBS, and dyed with reaction solution (C0071S, Beyotime, China). The images were collected with 100× visions in Nikon microscopy.

For colony formation assay, stable cells were cultured in six-well plates (1-2×10^3^ cells/well), and the medium was changed every three days. Fifteen days later, the cells were fixed in methanol for 30 min and stained with 0.1% crystal violet for 30 min. Colonies were photographed and counted.

**Metabolite measurements.**

Untargeted metabolome profiling was performed by Applied Protein Tech Co., Ltd (Shanghai, China). Targeted metabolome profiling was performed by (cells) Panomix Biomedical Tech Co., Ltd (Suzhou, China) and (tissues) Novogene Biomedical Tech Co., Ltd (Beijing, China). Cells and tissues were washed with 5% (wt/wt) mannitol and scraped with methanol in the presence of 10 µM of internal control solution comprising compound C1 with m/z at 182.048 and compound A1 with m/z at 231.070. Supernatants were filtered by centrifuge filter unit and were evaporated by centrifugation. Extracted intracellular metabolites were analyzed using a capillary electrophoresis-connected electrospray ionization/time-of-flight mass spectrometry and capillary electrophoresis tandem mass spectroscopy system.

**pH measurement of the cell culture media**

Same number of different groups of cells were seeded in the same dish and given the same amount of culture media. After a same incubation period (at least 48 h), the culture media of each group were collected and centrifuged at 1000 rpm for 5 min. The pH of supernatants was measured by a pH meter (METTLER TOLEDO, Delaware, USA) with the accuracy of 0.002. Mean pH of each group was calculated. In order to reduce the effect of cell proliferation, the seeding density was required to reach 90%.

**RNA-sequencing**

Total RNAs were isolated from HCC cells by Trizol reagent (Life Technologies, Carlsbad, California, USA) according to the manufacturer’s instructions. The amount and quantity of mRNA in the total RNA were tested by Nanodrop. The cDNA libraries were sequenced on the Illumina Hi-seq 2000 platform by Sagene Biotechnology Co., Ltd (Guangzhou, China). Please refer to Table S5.

**Glucose consumption assay**

For glucose consumption assay, glucose colorimetric assay kit was adopted (Bio Vision, U.S.A). HCC cells were first seeded in 6 cm dishes at a density of 2×10^6^ cells per dish and cultured with complete medium for 12 h in a 37 °C incubator. Then the medium was replaced with 3 ml complete medium. 24 h later, supernatants of HCC cells were collected and analyzed for glucose concentrations. The samples were measured at 570 nm and the glucose concentration was calculated based on previous established Standard Curve. Glucose consumption was extrapolated by subtracting the measured glucose concentrations in the medium from the original glucose concentration (25 mM).

**Supplementary figure legends**

**Figure S1. The expression of RFX6 in HCC public datasets.** (A) Comparison of RFX6 expression between paired HCC and normal tissues (n = 175) using ICGC data. (B) Comparison of RFX6 expression in HCC (n = 268) versus normal tissues (n = 289) using the GEO data (GSE25097). (C) Comparison of RFX6 expression in HCC (n = 240) versus normal tissues (n = 193) using the GEO data (GSE36376). (D) Comparison of RFX6 expression in HCC (n = 228) versus normal tissues (n = 168) using the GEO data (GSE63898). (E) Expression and subcellular localization of RFX6 detected by tyramide signal amplification. Scale bar, 5 μm. (F) Progression-free survival curves comparing patients with high versus low RFX6 expression using the TCGA data (n=365). (G) Overall survival curves comparing patients with high versus low RFX6 expression using the ICGC data (n=203). (H) Kaplan-Meier plots illustrating tumor doubling rate differences between patients with high and low RFX6 expression using the GEO data (GSE54236) (n=78). *P< 0.05, **P < 0.01, ***P< 0.001, and ****P< 0.0001.

**Figure S2. Silencing RFX6 suppresses proliferation and motility of HCC cells in vitro.** (A) Relative protein (top panel) and mRNA (bottom panel) levels of RFX6 in various HCC cell lines compared to the normal liver cell line, MIHA. (B) Western blotting (top panel) and real-time PCR (bottom panel) demonstrate the efficiency of RFX6-knockdown (KD). (C) Schematic illustration of RFX6-knockout achieved using the CRISPR-Cas9 system. (D) RFX6-KD suppressed the proliferation of HCC cells, as shown by the colony formation assay. (E) RFX6-KD suppressed the proliferation of HCC cells, as shown by the CCK-8 assay. (F) RFX6-KD suppressed the proliferation of HCC cells, as shown by the EDU assay. (G-H) RFX6-KD suppressed the migration (G) and invasion (H) capabilities of HCC cells, as shown by the transwell assay. 3.2×10^5^ cells were seeded in a chamber, and incubated for 12 h (migration assay) or 36 h (invasion assay) incubation. Data are represented as mean ± SD from at least three independent experiments. *P< 0.05, **P < 0.01, and ***P< 0.001.

**Figure S3. Overexpressing RFX6 promotes proliferation and motility of HCC cells in vitro and in vivo.** (A) RFX6-overexpressing (OE) promoted the proliferation of HCC cells, as shown by the colony formation assay. (B) RFX6-OE promoted the proliferation of HCC cells, as shown by the CCK-8 assay. (C) RFX6-OE promoted the proliferation of HCC cells, as shown by the EDU assay. (D) RFX6-OE promoted the migration and invasion capabilities of HCC cells, as shown by the transwell assay. 1.6×10^5^ cells were seeded in a chamber, and incubated for 12 h (migration assay) or 36 h (invasion assay) incubation. (E) RFX6-OE promoted HCC growth, as demonstrated by bioluminescent imaging (n=8/group). (F) RFX6-OE promoted HCC growth in vivo, as indicated by liver weights (n=6/group). Scale bar, 1 cm. (G) Hematoxylin/Eosin and immunohistochemical staining of RFX6, Ki67, and downstream protein in orthotopic tumors of mice. RFX6-OE promoted HCC growth, as indicated by the quantitation of Ki67+ stained cells. Data are represented as mean ± SD from at least three independent experiments. A two-tailed Student’s t test was used for statistical analysis. *P< 0.05, **P < 0.01, and ***P< 0.001.

**Figure S4. RFX6 promotes aerobic glycolysis in HCC.** (A) of metabolites from RFX6-knockout (KO) and control Hep3B cells, based on untargeted metabolome profiling. (B) GSEA plot illustrating that RFX6 expression is positively correlated with gene signatures related to the glycolysis and gluconeogenesis pathway, based on RNA-seq analysis of RFX6-KO and control Hep3B cells. (C) Analysis of the extracellular acidification rate (ECAR) in RFX6-KO and control Hep3B cells. (D) Analysis of the ECAR in RFX6-knockdown (KD) and control PLC/PRF/5 cells. (E) Analysis of the ECAR in RFX6-overexpressing (OE) and control Huh7 cells. (F) Analysis of the ECAR in RFX6-OE and control MHCC-97H cells. (G) PCA of metabolites from RFX6-KO and control Hep3B cells, based on targeted metabolome profiling of glucose metabolism. (H) Quantification of metabolic intermediates in RFX6-KO and control Hep3B cells using capillary electrophoresis-mass spectrometry. (I) Measurement of culture medium pH and lactate production in RFX6-KD and control PLC/PRF/5 cells. (J) Measurement of culture medium pH and lactate production in RFX6-OE and control MHCC-97H cells. Data are represented as mean ± SD from at least three independent experiments. *P< 0.05, **P < 0.01, ***P< 0.001, and ****P< 0.0001.

**Figure S5. Aerobic glycolysis is responsible for the progression of HCC induced by RFX6.** (A) Relative metabolite levels (left panel) and ratios (right panel) in glycolysis were measured and calculated from extracts of subcutaneous tumors in the RFX6-knockout (KO) and control groups (n=3/group). GLC, glucose; G6P, glucose 6-phosphate; PEP, phosphoenolpyruvate. (B) Relative metabolite levels (left panel) and ratios (right panel) in glycolysis were measured and calculated from extracts of subcutaneous tumors in the RFX6-overexpressing (OE) and control groups (n=3/group). (C-D) Lactate production in Huh7 (C) and MHCC-97H (D) cells was assessed after treatment with various concentrations of 2-DG. (E-F) 2-DG treatment counteracted the proliferation of Huh7 (E) and MHCC-97H (F) cells induced by RFX6, as shown by the CCK-8 assay. (G-H) 2-DG treatment counteracted the motility of Huh7 (G) and MHCC-97H (H) cells induced by RFX6, as shown by the transwell assay. 1.6×10^5^ cells were seeded in a chamber, and incubated for 12 h (migration assay) or 36 h (invasion assay) incubation. (I) The CCK-8 assay showed the effect of lactate supplementation on the proliferation of Hep3B cells. Data are represented as mean ± SD from at least three independent experiments. *P< 0.05, **P < 0.01, ***P< 0.001, and ****P< 0.0001.

**Figure S6. RFX6 transcriptionally regulates PGAM1.** (A) Expression of potential downstream targets was measured by real-time PCR (RT-PCR) in RFX6-knockdown (KD) HCC cells. (B) Expression of potential downstream targets was measured by RT-PCR in RFX6-overexpressing (OE) HCC cells. (C) RFX6 occupancy at the ADH5 promoter visualized by the Integrated Genomics Viewer. (D) Efficiency of HA-tagged RFX6 transfection confirmed by Western blotting. (E) ChIP-qPCR analysis of RFX6 occupancy at the ADH5 promoter region in Huh7 cells. IgG served as the control. (F) Correlation analysis of RFX6 and PGAM1 mRNA expression based on the TCGA data. (G) Correlation analysis of RFX6 and PGAM1 protein expression in HCC tissues from the SYSUCC cohort, based on IHC scores. (H) PGAM1-KD suppressed the proliferation of HCC cells, as shown by the colony formation assay. (I) PGAM1-KD suppressed the proliferation of HCC cells, as shown by the EDU assay. (J) PGAM1-KD suppressed the migration capability of HCC cells, as shown by the transwell assay. 3.2×10^5^ cells were seeded in a chamber, and incubated for 12 h (migration assay) or 36 h (invasion assay) incubation. (K) Western blotting demonstrates the efficiency of PGAM1-KD. (L) 2-PG production was measured in RFX6-KD and control PLC/PRF/5 cells. (M) 2-PG production was measured in RFX6-OE and control MHCC-97H cells. (N) The effect of 2-PG supplementation on cell proliferation was assessed using the CCK-8 assay. Data are represented as mean ± SD from at least three independent experiments. *P< 0.05, **P < 0.01, and ***P< 0.001.

**Figure S7. RFX6 promotes HCC progression via a PGAM1-dependent pathway.** (A) Real-time PCR demonstrates the efficiency of transient PGAM-knockdown (KD) in RFX6-overexpressing (OE) Huh7 cells. (B) Western blotting demonstrates the efficiency of transient PGAM1-KD in RFX6-OE and control Huh7 cells. (C) Stable PGAM1-KD attenuated the RFX6-induced proliferation of HCC cells, as shown by the colony formation assay. (D-E) Transient PGAM1-KD attenuated the RFX6-induced proliferation of HCC cells, as shown by the colony formation assay. (F) Transient PGAM1-KD attenuated the RFX6-induced proliferation of HCC cells, as shown by the CCK-8 assay. (G) Stable PGAM1-KD attenuated the RFX6-induced motility of HCC cells, as shown by the transwell assay. (H) Transient PGAM1-KD attenuated the RFX6-induced proliferation of HCC cells, as shown by the EDU assay. (I) Transient PGAM1-KD attenuated the RFX6-induced motility of HCC cells, as shown by the transwell assay. 1.6×10^5^ cells were seeded in a chamber, and incubated for 12 h (migration assay) or 36 h (invasion assay) incubation. (J-K) Transient PGAM1-KD attenuated the RFX6-induced glycolysis in HCC cells, as determined by Seahorse experiments (J), lactate production assay, and 2-PG production assay (K). Data are represented as mean ± SD from at least three independent experiments. *P< 0.05, **P < 0.01, ***P< 0.001, and ****P< 0.0001.

**Figure S8. Overexpressing PGAM1 reverses the inhibited cell proliferation, motility, and glycolysis induced by silencing RFX6.** (A) Western blotting demonstrates the efficiency of PGAM1-overexpressing (OE) in RFX6-Knockout (KO) Hep3B cells. (B) PGAM1-OE enhanced the RFX6-KO induced-cell proliferation, as shown by the colony formation assay. (C) PGAM1-OE enhanced the RFX6-KO induced-cell proliferation, as shown by the CCK-8 assay. (D) PGAM1-OE enhanced RFX6-KO induced-cell proliferation as shown by EDU assay. (E) PGAM1-OE enhanced the RFX6-KO induced-cell motility, as shown by the transwell assay. 3.2×10^5^ cells were seeded in a chamber, and incubated for 12 h (migration assay) or 36 h (invasion assay) incubation. (F) PGAM1-OE enhanced the RFX6-KO induced-glycolysis, as determined by pH measurements, lactate production assay, and 2-PG production assay. (G) PGAM1-OE enhanced the RFX6-KO induced-glycolysis, as determined by Seahorse experiments. Data are represented as mean ± SD from at least three independent experiments. *P< 0.05, **P < 0.01, ***P< 0.001, and ****P< 0.0001.
