# Supplementary material for: RFX6 facilitates aerobic glycolysis‐mediated growth and metastasis of hepatocellular carcinoma through targeting PGAM1
Source: Clin Transl Med. 2023 Dec 13;13(12):e1511. doi: 10.1002/ctm2.1511 (PMC10719540; doi:10.1002/ctm2.1511)
Supplement: Supplementary file 10 — Supporting Information [file CTM2-13-e1511-s003.docx]

**Table S1.**

Demographic and clinical characteristics of the enrolled HCC patients used in microarray cohort.

| Variable | RFX6 Low | RFX6 High | P value |
| --- | --- | --- | --- |
|  | (n = 60) | (n = 65) |  |
| Gender (Male) | 54 (90.0) | 56 (86.2) | 0.509 |
| age (≥54) | 29 (48.3) | 39 (60.0) | 0.191 |
| HBV infection (yes) | 58 (96.7) | 60 (92.3) | 0.503 |
| HBV-DNA (High) | 40 (66.7) | 38 (59.4) | 0.401 |
| ALT (>50 U/L) | 16 (26.7) | 16 (24.6) | 0.793 |
| AST (>40 U/L) | 27 (45.0) | 22 (33.8) | 0.202 |
| ALP (>125 U/L) | 14 (23.3) | 13 (20.0) | 0.651 |
| ALB (>35 g/L) | 54 (90.0) | 61 (93.8) | 0.644 |
| TBIL (>17.1 µmol/L) | 10 (16.7) | 17 (26.2) | 0.198 |
| GGT (>60 U/L) | 28 (46.7) | 30 (46.2) | 0.954 |
| AFP (>200 ng/mL) | 17 (28.3) | 21 (32.3) | 0.629 |
| Cirrhosis (yes) | 37 (61.7) | 41 (63.1) | 0.871 |
| Microvascular invasion (yes) | 19 (31.7) | 18 (27.7) | 0.627 |
| Multiple tumors (yes) | 25 (5.0) | 9 (13.8) | 0.093 |
| Tumor size (>5 cm) | 31 (51.7) | 30 (46.2) | 0.195 |
| Adjacent organ invasion (yes) | 6 (10.0) | 16 (24.6) | **0.032** |
| Tumor capsule (no) | 20 (33.3) | 33 (50.8) | **0.049** |
| Vascular invasion (yes) | 15 (25.0) | 36 (48.0) | **0.006** |
| Lymph node metastasis (yes) | 5 (8.3) | 15 (23.1) | **0.028** |
| Resection margin (<1cm) | 4 (6.7) | 17 (26.2) | **0.004** |

Values are presented as n (%).

Abbreviation:

HBV: hepatitis B virus. ALT: alanine aminotransferase. AST: aspartate aminotransferase. ALP: alkaline phosphatase. ALB: albumin. TBIL: total bilirubin. GGT: γ-glutamyl transpeptidase. AFP: alpha-fetoprotein.

**Table S2.**

Univariate and multivariate cox regression analyses of the prognostic factors for overall survival.

| Variable | Univariate analysis | | Multivariate analysis | |
| --- | --- | --- | --- | --- |
|  | HR (95% CI) | P value | HR (95% CI) | P value |
| Gender (Female/Male) | 2.092 (0.64-6.81) | 0.221 |  |  |
| Age, y (<54/≥54) | 1.106 (0.59-2.07) | 0.751 |  |  |
| HBV infection (no/yes) | 0.419 (0.15-1.19) | 0.103 |  |  |
| HBV-DNA(Normal/High) | 1.178 (0.61-2.29) | 0.631 |  |  |
| ALT, U/L (≤50/>50) | 1.253 (0.63-2.51) | 0.525 |  |  |
| AST, U/L (≤40/>40) | 1.461 (0.78-2.74) | 0.237 |  |  |
| ALP, U/L (≤125/>125) | 1.030 (0.47-2.24) | 0.942 |  |  |
| ALB, g/L (≥35/<35) | 1.118 (0.34-3.63) | 0.853 |  |  |
| TBIL, µmol/L (≤17.1/>17.1) | 1.091 (0.51-2.32) | 0.822 |  |  |
| GGT, U/L (≤60/>60) | 1.339 (0.72-2.50) | 0.359 |  |  |
| AFP, ng/mL (≤200/>200) | 0.880 (0.45-1.73) | 0.711 |  |  |
| Resection margin (≥1/<1 cm) | 5.399 (2.39-12.20) | **< 0.001** |  | 0.066 |
| Cirrhosis(no/yes) | 0.999 (0.52-1.91) | 0.997 |  |  |
| Multiple tumors (no/yes) | 2.871 (1.35-6.09) | **0.006** | 2.217 (1.00-4.90) | **0.049** |
| Tumor size (≤5/>5 cm) | 0.619 (0.31-1.22) | 0.166 |  |  |
| Adjacent Organ Invasion (no/yes) | 39.583 (16.91-92.63) | **< 0.001** | 43.921 (13.27-145.36) | **< 0.001** |
| Microvascular invasion (no/yes) | 1.796 (0.95-3.41) | **0.074** |  | 0.089 |
| Tumor encapsule (yes/no) | 1.619 (0.87-3.02) | 0.130 |  |  |
| Macrovascular invasion (no/yes) | 5.903 (3.08-11.31) | **< 0.001** |  | 0.182 |
| Lymph node metastasis (no/yes) | 2.671 (1.16-6.14) | **0.021** |  | 0.804 |
| RFX6 (Low/High) | 5.805 (2.56-13.16) | **<0.001** | 3.072 (1.28-7.40) | **0.012** |

Abbreviation:

HR: hazard ratio. CI: confidence interval. HBV: hepatitis B virus. ALT: alanine aminotransferase. AST: aspartate aminotransferase. ALP: alkaline phosphatase. ALB: albumin. TBIL: total bilirubin. GGT: γ-glutamyl transpeptidase. AFP: alpha-fetoprotein.

**Table S3.**

Univariate and multivariate cox regression analyses of the prognostic factors for disease free survival.

| Variable | Univariate analysis | | Multivariate analysis | |
| --- | --- | --- | --- | --- |
|  | HR (95% CI) | P value | HR (95% CI) | P value |
| Gender (Female/Male) | 1.282 (0.58-2.82) | 0.537 |  |  |
| Age, y (<54/≥54) | 0.936 (0.57-1.54) | 0.795 |  |  |
| HBV infection (no/yes) | 1.065 (0.39-2.94) | 0.903 |  |  |
| HBV-DNA(Normal/High) | 0.963 (0.57-1.62) | 0.887 |  |  |
| ALT, U/L (≤50/>50) | 0.978 (0.55-1.73) | 0.940 |  |  |
| AST, U/L (≤40/>40) | 1.243 (0.75-2.06) | 0.399 |  |  |
| ALP, U/L (≤125/>125) | 1.523 (0.85-2.73) | 0.158 |  |  |
| ALB, g/L (≥35/<35) | 1.080 (0.43-2.71) | 0.870 |  |  |
| TBIL, µmol/L (≤17.1/>17.1) | 1.145 (0.63-2.08) | 0.657 |  |  |
| GGT, U/L (≤60/>60) | 1.306 (0.79-2.15) | 0.295 |  |  |
| AFP, ng/mL (≤200/>200) | 0.980 (0.57-1.69) | 0.942 |  |  |
| Resection margin (≥1/<1 cm) | 1.938 (1.04-3.62) | **0.038** |  | 0.390 |
| Cirrhosis(no/yes) | 1.223 (0.72-2.07) | 0.453 |  |  |
| Multiple tumors (no/yes) | 2.776 (1.43-5.39) | **0.003** |  | 0.062 |
| Tumor size (≤5/>5 cm) | 0.870 (0.53-1.44) | 0.589 |  |  |
| Adjacent organ invasion (no/yes) | 4.994 (2.76-9.03) | **<0.001** | 3.766 (2.02-7.03) | **<0.001** |
| Microvascular invasion (no/yes) | 1.272 (0.74-2.19) | 0.384 |  |  |
| Tumor encapsule (yes/no) | 1.137 (0.69-1.88) | 0.615 |  |  |
| Macrovascular invasion (no/yes) | 2.497 (1.46-4.27) | **0.001** |  | 0.669 |
| Lymph node metastasis (no/yes) | 1.479 (0.76-2.87) | 0.247 |  |  |
| RFX6 (Low/High) | 2.379 (1.39-4.08) | **0.002** | 1.806 (1.02-3.19) | **0.042** |

Abbreviation:

HR: hazard ratio. CI: confidence interval. HBV: hepatitis B virus. ALT: alanine aminotransferase. AST: aspartate aminotransferase. ALP: alkaline phosphatase. ALB: albumin. TBIL: total bilirubin. GGT: γ-glutamyl transpeptidase. AFP: alpha-fetoprotein.
