# Supplementary material for: RFX6 facilitates aerobic glycolysis‐mediated growth and metastasis of hepatocellular carcinoma through targeting PGAM1
Source: Clin Transl Med. 2023 Dec 13;13(12):e1511. doi: 10.1002/ctm2.1511 (PMC10719540; doi:10.1002/ctm2.1511)
Supplement: Supplementary file 13 — Supporting Information [file CTM2-13-e1511-s013.docx]

**Table S6 The top 10 enriched pathways for RFX6 ChIP-seq peaks (ranked by P value)**

| Term | Enrichment | P value | FDR | Gene number |
| --- | --- | --- | --- | --- |
| Hippo signaling pathway | 1.835 | 2.51E-07 | 7.94E-05 | 154 |
| **Metabolic pathways** | **1.336** | **5.27E-07** | **8.33E-05** | **1248** |
| Pathways in cancer | 1.400 | 5.65E-06 | 0.000595363 | 530 |
| Focal adhesion | 1.677 | 1.52E-05 | 0.001202524 | 199 |
| MAPK signaling pathway | 1.526 | 2.10E-05 | 0.001327411 | 295 |
| Axon guidance | 1.796 | 2.71E-05 | 0.001428379 | 181 |
| Hepatocellular carcinoma | 1.638 | 5.08E-05 | 0.002291786 | 168 |
| Regulation of actin cytoskeleton | 1.742 | 9.40E-05 | 0.003598399 | 214 |
| Colorectal cancer | 1.930 | 0.000102486 | 0.003598399 | 86 |

Abbreviation:

ChIP: Chromatin immunoprecipitation. MAPK: mitogen-activated protein kinase. FDR: false discovery rate.

* Only the pathways with P values <0.05 are considered.
