# Supplementary material for: RFX6 facilitates aerobic glycolysis‐mediated growth and metastasis of hepatocellular carcinoma through targeting PGAM1
Source: Clin Transl Med. 2023 Dec 13;13(12):e1511. doi: 10.1002/ctm2.1511 (PMC10719540; doi:10.1002/ctm2.1511)
Supplement: Supplementary file 16 — Supporting Information [file CTM2-13-e1511-s002.docx]

**Table S9.**

Primer sequences for RT-PCR and ChIP-qPCR used in current study.

| Gene | Primer sequence (5’-3’) |
| --- | --- |
| **RT-PCR primers** |  |
| RFX6 Forward (5’-3’) | ACTCACTGCCAGTGTATCCTGG |
| RFX6 Reverse (5’-3’) | GGAAGGAGATGGTCAGGCATTC |
| GAPDH Forward (5’-3’) | GTCTCCTCTGACTTCAACAGCG |
| GAPDH Reverse (5’-3’)  ADH5 Forward (5’-3’) | ACCACCCTGTTGCTGTAGCCAA  GTAAACCCATCCAGGAAGTGCTC |
| ADH5 Reverse (5’-3’) | TGTGACATGCCTCAAGTGCTGC |
| PGAM1 Forward (5’-3’) | GCTCTGCCCTTCTGGAATGAAG |
| PGAM1 Reverse (5’-3’) | ATACCAGTCGGCAGGTTCAGCT |
| **ChIP-PCR primers** |  |
| PGAM1 Forward (5’-3’) | AGGAGGGAAACCTGGCTGTG |
| PGAM1 Reverse (5’-3’)  ADH5 Forward (5’-3’)  ADH5 Reverse (5’-3’) | TGAGTAATGAAAGGGGAAAACG  CTCTCCCTATGACGAGTCCCT  TGAAAGCGCCTGAGAACCAG |

Abbreviation:

RT-PCR: real-time PCR. ChIP: Chromatin immunoprecipitation. qPCR: quantitative PCR.

**Table S10.**

Antibodies included in current study.

| Antibody | Company | Catalog no. | Dilution |
| --- | --- | --- | --- |
| **Western blotting** | | | |
| RFX6 | Proteintech | 22551-1-AP | 1:500 |
| α-tubulin | Proteintech | 66031-1-Ig | 1:2000 |
| ADH5 | Abcam | ab175406 | 1:1000 |
| GAPDH | Proteintech | 60004-1-Ig | 1:2000 |
| PGAM1 | Novus | NBP1-49532 | 1:1000 |
| β-actin | Cell Signaling Technology | 3700 | 1:1000 |
| HA | Abcam | ab9110 | 1:5000 |
| Anti-mouse IgG | Cell Signaling Technology | 7076s | 1:3000 |
| Anti-rabbit IgG | Cell Signaling Technology | 7074s | 1:3000 |
| **Immunohistochemical staining** | | | |
| RFX6 | Proteintech | 22551-1-AP | 1:250 |
| PGAM1 | Novus | NBP1-49532 | 1:3000 |
| Ki67 | Abcam | ab16667 | 1:800 |
| **Chromatin immunoprecipitation** | | | |
| HA | Abcam | ab9110 | 1:5000 |
| Normal rabbit IgG | Cell Signaling Technology | 2729s | - |
| Histone H3 | Cell Signaling Technology | 4499s | - |
